# Identification of a new cannabidiol n-hexyl homolog in a medicinal cannabis variety with an antinociceptive activity in mice: cannabidihexol

**DOI:** 10.1038/s41598-020-79042-2

**Published:** 2020-12-16

**Authors:** Pasquale Linciano, Cinzia Citti, Fabiana Russo, Francesco Tolomeo, Aldo Laganà, Anna Laura Capriotti, Livio Luongo, Monica Iannotta, Carmela Belardo, Sabatino Maione, Flavio Forni, Maria Angela Vandelli, Giuseppe Gigli, Giuseppe Cannazza

**Affiliations:** 1 Department of Life Sciences, University of Modena and Reggio Emilia, Via Campi 103, 41125 Modena, Italy; 2 Mediteknology (CNR Spin-Off Company), Via Arnesano, 73100 Lecce, Italy; 3 CNR NANOTEC, Istituto di Nanotecnologia, Via Monteroni, 73100 Lecce, Italy; 4 Department of Chemistry, Sapienza University of Rome, Piazzale Aldo Moro 5, 00185 Rome, Italy; 5 Division of Pharmacology, Università degli studi della Campania “L. Vanvitelli”, Via Costantinopoli, 16, 80138 Naples, Italy

**Keywords:** Analytical chemistry, Medicinal chemistry, Organic chemistry, Chemistry, Asymmetric synthesis, Natural product synthesis

## Abstract

The two most important and studied phytocannabinoids present in *Cannabis sativa* L. are undoubtedly cannabidiol (CBD), a non-psychotropic compound, but with other pharmacological properties, and Δ^9^-tetrahydrocannabinol (Δ^9^-THC), which instead possesses psychotropic activity and is responsible for the recreative use of hemp. Recently, the homolog series of both CBDs and THCs has been expanded by the isolation in a medicinal cannabis variety of four new phytocannabinoids possessing on the resorcinyl moiety a butyl-(in CBDB and Δ^9^-THCB) and a heptyl-(in CBDP and Δ^9^-THCP) aliphatic chain. In this work we report a new series of phytocannabinoids that fills the gap between the pentyl and heptyl homologs of CBD and Δ^9^-THC, bearing a *n*-hexyl side chain on the resorcinyl moiety that we named cannabidihexol (CBDH) and Δ^9^-tetrahydrocannabihexol (Δ^9^-THCH), respectively. However, some cannabinoids with the same molecular formula and molecular weight of CBDH and Δ^9^-THCH have been already identified and reported as monomethyl ether derivatives of the canonical phytocannabinoids, namely cannabigerol monomethyl ether (CBGM), cannabidiol monomethyl ether (CBDM) and Δ^9^-tetrahydrocannabinol monomethyl ether (Δ9-THCM). The unambiguously identification in cannabis extract of the *n*-hexyl homologues of CBD and Δ^9^-THC different from the corresponding methylated isomers (CBDM, CBGM and Δ^9^-THCM) was achieved by comparison of the retention time, molecular ion, and fragmentation spectra with those of the authentic standards obtained via stereoselective synthesis, and a semi-quantification of these cannabinoids in the FM2 medical cannabis variety was provided. Conversely, no trace of Δ^9^-THCM was detected. Moreover, CBDH was isolated by semipreparative HPLC and its identity was confirmed by comparison with the spectroscopic data of the corresponding synthetic standard. Thus, the proper recognition of CBDH, CBDM and Δ^9^-THCH closes the loop and might serve in the future for researchers to distinguish between these phytocannabinoids isomers that show a very similar analytical behaviour. Lastly, CBDH was assessed for biological tests in vivo showing interesting analgesic activity at low doses in mice.

## Introduction

Cannabis research has made great progresses in the latest years in both clinical and academic field. For example, new cannabis based drugs, like Epidiolex, have been placed on the market for the treatment of severe forms of infant epilepsy not responding to conventional therapies^1^. In the academic research, new insights on cannabis chemistry have been disclosed thanks to the high-performing technological platforms for the identification of new compounds^2–6^. Although there is still much to do in the cannabis chemistry research, almost 150 phytocannabinoids can be count on the most updated inventory^7^. Our most recent works have disclosed the existence of new phytocannabinoids series besides those of the orcinoids, varinoids and olivetoids, belonging to the cannabidiol (CBD) and Δ^9^-tetrahydrocannabinol (Δ^9^-THC) type cannabinoids^4,8^. The new series of phytocannabinoids share the terpenophenolic core of CBD and Δ^9^-THC and differ for the length of the linear alkyl side chain; specifically, cannabidibutol (CBDB), and Δ^9^-tetrahydrocannabutol (Δ^9^-THCB) have a *n*-butyl side chain^8^, whereas cannabidiphorol (CBDP) and Δ^9^-tetrahydrocannabiphorol (Δ^9^-THCP) have a *n*-heptyl side chain^4^. The discovery of new phytocannabinoids, which were both directly isolated from the plant and synthetically prepared in the lab, has opened new gaps on their still unexplored biological activity, making us wondering about their pharmacological effects on humans.

To further complicate the already intricate scenario, we report a new series of phytocannabinoids that fills the gap between the pentyl and heptyl homologs of CBD and Δ^9^-THC, bearing a *n*-hexyl side chain on the resorcinyl moiety. At the best of our knowledge and according to the literature, no case of hexyl derivatives of cannabinoid has been reported so far. Conversely, cannabinoids with the same molecular formula and molecular weight have been classified as monomethyl ether derivatives of canonical phytocannabinoids, namely cannabigerol monomethyl ether (CBGM), cannabidiol monomethyl ether (CBDM) and Δ^9^-tetrahydrocannabinol monomethyl ether (Δ^9^-THCM)^9^. Whilst CBGM and CBDM have been already isolated and characterized^10,11^, Δ^9^-THCM has been detected in cannabis smoke^12^ and some authors reported that it is present in the plant, but they were not able to isolate it due to chromatographic issues^13^. Our findings on the presence of the hexyl homologs of CBD and Δ^9^-THC, which we named cannabidihexol (CBDH) and Δ^9^-tetrahydrocannabihexol (Δ^9^-THCH) respectively, were supported by the stereoselective synthesis of the corresponding pure standards that are found in the plant prior to decarboxylation.

## Results

### Identification of CBD and Δ^9^-THC hexyl homologs by UHPLC-HESI-Orbitrap

**Figure 1:**
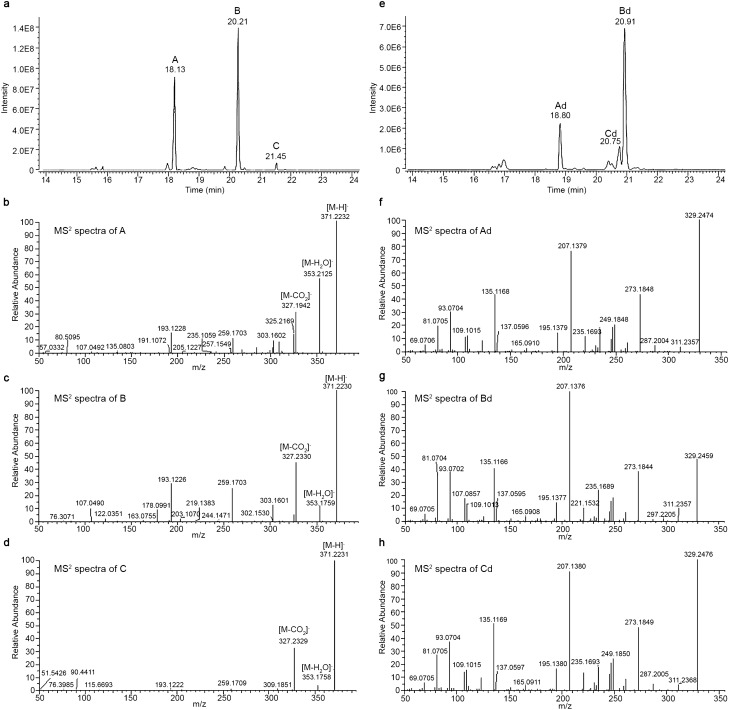
Identification of compounds corresponding to the molecular formula C_23_H_32_O_4_ in C. sativa FM2. (**A**) UHPLC-HRMS extracted ion chromatogram (EIC) for molecular formula C_23_H_32_O_4_ in native FM2 and the relative fragmentation spectra, in negative ionization mode, for the identified peaks A (panel **B**), B (panel **C**) and C (panel **D**). (**E**) UHPLC-HRMS extracted ion chromatogram (EIC) for molecular formula C_22_H_31_O_2_ in decarboxylated FM2 and the relative fragmentation spectra, in negative ionization mode, for the identified peaks Ad (panel **F**), Bd (panel **G**) and Cd (panel **H**).

In the attempt to provide a complete characterization of the FM2 cannabis variety, we noticed the presence of two major peaks at 18.13 and 20.21, and a minor one at 21.46 min, corresponding to the molecular formula C_23_H_32_O_4_, suggesting the presence of the carboxylic group. The analysis of the fragmentation spectra in negative ionization mode confirmed this hypothesis but showed three different fragmentation patterns (Fig. 1A). The two major peaks A and B presented very similar spectra differing only for the relative intensity of the fragments, whereas the minor peak C showed very poor fragmentation. Peak A at 18.13 min could be associated to a CBDA-like molecule (Fig. 1B), while peak B at 20.21 min had lower intensity for the fragment corresponding to [M–H_2_O]^−^ at *m*/*z* 353 and presented a new fragment at *m*/*z* 178, not found in the other spectra (Fig. 1C). Peak C at 21.46 min showed a THCA-like fragmentation characterized by the very low intensity of the fragment [M–H_2_O]^−^ and the absence of other major fragments besides the one corresponding to [M-CO_2_]^−^ at *m*/*z* 327 (Fig. 1D).

In order to identify these compounds, but being unable to isolate acidic species, we moved to work on the decarboxylated forms of such cannabinoids. Therefore, the ethanolic extract of FM2 was heated and the new mixture was analysed employing the same conditions by UHPLC-HESI-Orbitrap. As expected, in place of the previously detected peaks, three new peaks appeared at different retention times, 18.62, 20.62, and 20.77 min, with the molecular formula C_22_H_32_O_2_ corresponding exactly to the loss of a CO_2_ molecule. Figure 1E shows a second chromatogram with the decarboxylated compounds A_d_, B_d_, and C_d_. Surprisingly, peaks B_d_ and C_d_ had inverted elution order and peaks A_d_ and C_d_ presented superimposable fragmentation spectra in positive ionization mode with the same pattern as CBD and THC. Moreover, peaks A_d_ and B_d_ were very similar and differed for the relative intensity of the molecular ion [M+H]^+^ at *m*/*z* 329 and the base peak at *m*/*z* 207. We concluded that peak A and B could be acidic CBD-type cannabinoids, whereas peak C could be an acidic THC-type cannabinoid (Fig. 1F–H).

**Figure 2:**
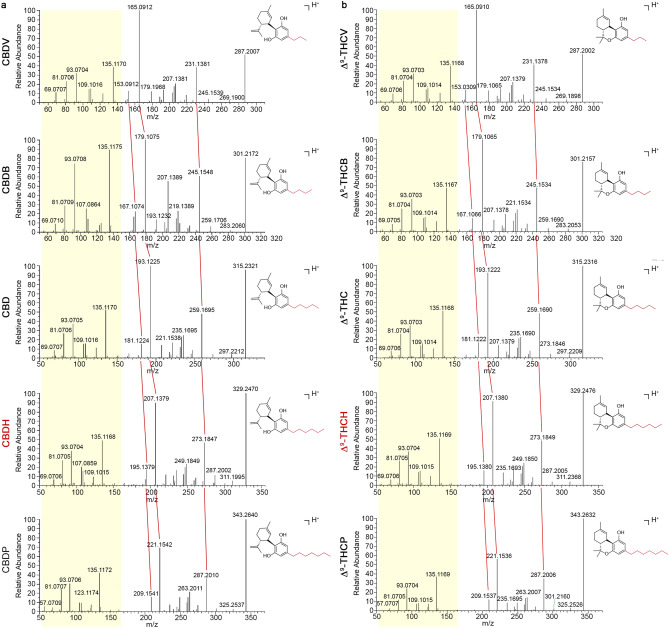
MS/MS spectra library of CBD and Δ^9^-THC homologs by UHPLC-HESI-Orbitrap. Comparison of the high-resolution fragmentation spectra in positive (ESI+) mode for CBD (panel **A**) and Δ^9^-THC (panel **B**) homologs. The pale-yellow box point out the constant terpenic portion. The red lines highlight the shift of some fragments corresponding to the loss of a methylene portion (CH_2_, m/z = 14) moving from CBDP (or Δ^9^-THCP) to CBDV (or Δ^9^-THCV).

According to the literature, cannabinoids with such molecular formula and molecular ions are reported as monomethyl ethers of CBDA and THCA, named cannabidiolic acid monomethyl ether (CBDMA) and tetrahydrocannabinolic acid monomethyl ether (THCMA). Similarly, cannabinoids with molecular formula C_22_H_32_O_2_ could be the corresponding decarboxylated derivatives, the already known cannabidiol monomethyl ether (CBDM) and the putative tetrahydrocannabinol monomethyl ether (THCM). However, we found three peaks corresponding to the same formula but different MS^2^ spectra. By a comparison with other CBD and THC homologs present in our spectral library, such as cannabidivarin (CBDV), cannabidibutol (CBDB), cannabidiphorol (CBDP), Δ^9^-tetrahydrocannabivarin (Δ^9^-THCV), Δ^9^-tetrahydrocannabutol (Δ^9^-THCB), and Δ^9^-tetrahydrocannabiphorol (Δ^9^-THCP), we were able to putatively identify two new homologs of CBD and THC with a hexyl side chain. As shown in Fig. 2, the new compounds differ exactly by a –CH_2_ unit (14 amu) from the corresponding pentyl (CBD and Δ^9^-THC) and the recently identified heptyl homologs (CBDP and THCP), not only for the molecular ion [M+H]^+^ but also for all fragments. For both CBD (Fig. 2A) and THC (Fig. 2B) homologs, it was evident that the molecular ion [M+H]^+^ and the base peak inverted their relative intensity as the length of the side chain increased from the propyl to the heptyl homologs, most likely due to the increasing stability of the molecular ion.

In order to confirm the identity of these two new cannabinoids and unambiguously identify peak B_d_, a stereoselective synthesis of the putatively identified cannabinoids, which for sake of simplicity and consistency were called cannabidihexol (CBDH) and Δ^9^-tetrahydrocannabihexol (Δ^9^-THCH), and the monomethyl ether derivatives of CBD and Δ^9^-THC (CBDM and Δ^9^-THCM) was performed.

### Identification of CBGM by UHPLC-HESI-Orbitrap

Given the results obtained for CBD and Δ^9^-THC, we hypothesized the presence in the FM2 variety of the hexyl homolog of CBG, which has a molecular formula C_22_H_34_O_2_ and [M+H]^+^ ion at *m*/*z* 331.2632. Only one peak resulted from the specific ion search, thus instilling the doubt about its identity as hexyl or methyl ether derivative. The fragmentation spectrum of its acidic precursor in the FM2 native extract showed a pattern different from that of cannabigerolic acid (CBGA), for which the analytical standard was available, and from those reported in the literature for the other series of CBG like cannabigerovarin (CBGV)^14^ and cannabigerobutol (CBGB)^9^. The match of retention time (20.98 min) and fragmentation spectrum with those of synthetic CBGM and the comparison with spectral data reported in the literature for the same cannabinoid confirmed that the additional methyl group was attached to the oxygen of the resorcinyl moiety and was not part of the alkyl side chain.

### Stereoselective synthesis of CBDH, Δ^9^-THCH and monomethyl derivatives CBDM, Δ^9^-THCM and CBGM

**Figure 3:**
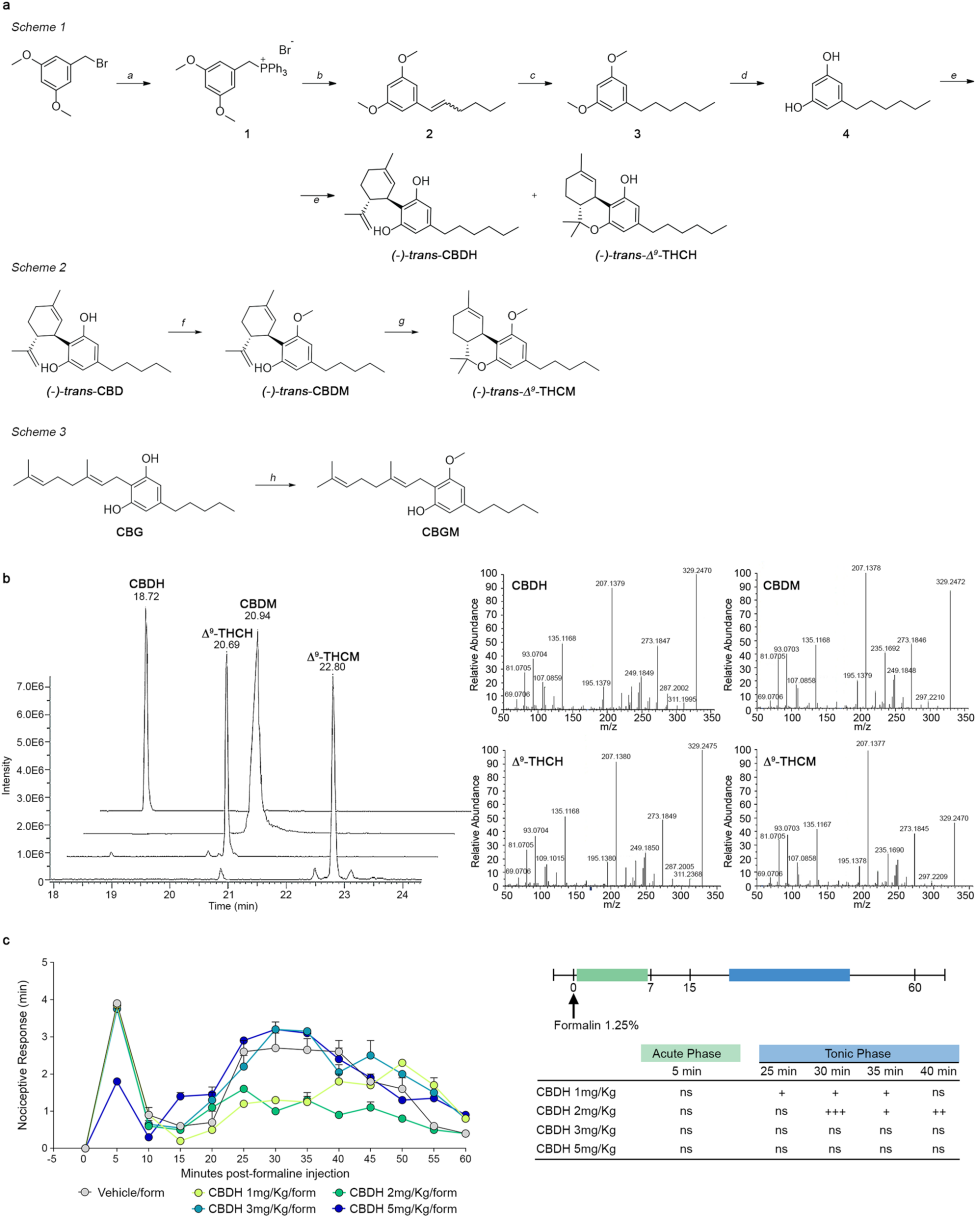
Synthesis and UHPLC-HRMS identification of CBDH, CBDM, Δ^9^-THCH and Δ^9^-THCM, and in vivo activity of CBDH. (**A**) *Scheme 1*. Reagents and conditions: (a) triphenylphosphine (1.1 equiv.), toluene, reflux, 6 h, quant. yield; (b) valeraldehyde (1.5 equiv.), 0.1 M K_2_CO_3_ aq. (10 mL *per* mmol of **1**), reflux, 5 h, 81% yield; (c) H-Cube ThalesNano H_2_-Pd/C, EtOH, 30 °C, 20 bar, 1 mL/min, 91% yield; (d) BBr_3_ 1 M in DCM (2.2 eq.), anhydrous DCM, N_2_ atmosphere, − 15 °C → r.t, 24 h, quant. yield; (e) (1*S*,4*R*)-1-methyl-4-(prop-1-en-2-yl)cycloex-2-enol (0.9 equiv.), pTSA (0.1 equiv.), DCM, r.t., argon, 2 h, 17% yield for (−)-*trans*-CBDH and 20% yield for (−)-*trans*-Δ^9^-THCH. *Scheme 2.* Reagents and conditions: dimethylsulphate (0.5 equiv.), K_2_CO_3_ (1 equiv.), DMF, r.t. 62% yield for (−)-trans-CBDM and 57% yield for CBGM. *Scheme 3.* Reagents and conditions: *p*-TSA (0.1 equiv.), dry DCM, r.t., 1 h, 43% yield. (**B**) Superimposition of extracted UHPLC-HRMS ion chromatograms (EICs) of synthetic cannabinoid n-hexyl and monomethyl ether homologs. and relative fragmentation spectra, in positive ionization mode. EICs were chosen based on the exact mass calculated for C_23_H_32_O_4_. (**C**) Effects of CBDH (1, 2, 3, and 5 mg/kg, i.p.) or vehicle in the formalin test in mice. The total time of the nociceptive response was measured every 5 min and expressed in min (see “Experimental” section). Data are represented as means ± SEM (n = 5–6). ^+,+++^indicate statistically significant differences versus veh/form, *p* < 0.05 and *p* < 0.001, respectively. 2-way ANOVA followed by Bonferroni’s post hoc tests was used for statistical analysis.

The stereoselective synthesis of (−)-trans-cannabidihexol ((−)-trans-CBDH) and (−)-trans-Δ^9^-tetrahydrocannabihexol ((−)-*trans*-Δ^9^-THCH) was performed as previously reported for the synthesis of the corresponding homologs (−)-*trans*-CBDB, (−)-*trans*-CBDP, (−)-*trans*-Δ^9^-THCB and (−)-*trans*-Δ^9^-THCP^2–4,8^. The appropriate 5-hexylbenzene-1,3-diol (**4**) was prepared first as reported in Fig. 3A, Scheme 1. (3,5-dimethoxybenzyl)triphenylphosphonium bromide (**1**) was easily prepared in quantitative yield by reaction of 1-(bromomethyl)-3,5-dimethoxybenzene with triphenylphosphine in refluxing toluene for 6 h. Wittig’s reaction between **1** and valeraldehyde in 0.1 M K_2_CO_3_ aqueous solution at reflux for 24 h gave 1-(hex-1-en-1-yl)-3,5-dimethoxybenzene (**2**) as a 55:45 E/Z mixture which was hydrogenated using the ThalesNano H-Cube flow reactor, to give the corresponding 1-hexyl-3,5-dimethoxybenzene (**3**) in 91% yield. The demethylation performed using BBr_3_ in anhydrous DCM, overnight at room temperature and under nitrogen atmosphere, gave 5-hexyl-resorcinol (**4**) in quantitative yield. In our previous works CBDB and CBDP were synthesized first by condensation of the appropriate resorcinol with (1S,4R)-1-methyl-4-(prop-1-en-2-yl)cycloex-2-enol, using *p*TSA as catalyst and stopping the reaction before CBDs evolve to THCs in the same conditions. Conversely, for the selective synthesis of (−)-*trans*-Δ^9^-THCB and (−)-*trans*-Δ^9^-THCP a longer procedure was adopted. The appropriate resorcinol was condensed with (1*S*,4*R*)-1-methyl-4-(prop-1-en-2-yl)cycloex-2-enol in the same condition described above for a longer reaction time (usually 48 h). In this way, the CBDs were quantitatively converted into the corresponding Δ^8^-THCs. Hydrochlorination of the Δ^8^ double bond of (−)-*trans*-Δ^8^-THCs, allowed to obtain (−)-*trans*-HCl-THCs, which were successively converted to (−)-*trans*-Δ^9^-THCs by selective elimination on position 2 of the terpene moiety using potassium *t*-amylate as base. Although this procedure allowed to selectively prepare (−)-*trans*-Δ^9^-THCs, it has the inconvenience to be time consuming, and with low atom economy. Because the conversion of CBDs to Δ^8^-THCs passes through the formation of Δ^9^-THCs first, for the synthesis of (−)-*trans*-CBDH and (−)-*trans*-Δ^9^-THCH we evaluated the possibility to stop the reaction before Δ^9^-THCH starts to convert into Δ^8^-THCH. Therefore, 5-hexyl-resorcinol (**4**) was condensed with (1S,4R)-1-methyl-4-(prop-1-en-2-yl)cycloex-2-enol using *p*TSA as catalyst and the progression of the reaction was monitored every 15 min by HPLC–UV/Vis. After approximately 2 h, almost the 50% of (−)-*trans*-CBDH converted in (−)-*trans*-Δ^9^-THCH, but no traces of Δ^8^-THCH were detected. The reaction was therefore stopped and (−)-*trans*-CBDH and (−)-*trans*-Δ^9^-THCH were purified as reported in Material and Methods section. (−)-*trans*-CBDH and (−)-*trans*-Δ^9^-THCH were obtained in 17% and 20% yield, respectively. The total yield of the two phytocannabinoids was 37%, which is in line with the yield obtained for the sole synthesis of CBDB or CBDP using the same procedure, but quenching the reaction after the consumption of the starting materials and before that CBDs started to isomerize into THCs (usually 30 min–1 h). Therefore, strictly monitoring the condensation of the appropriate resorcinol with (1S,4R)-1-methyl-4-(prop-1-en-2-yl)cycloex-2-enol it is possible to prepare in one-pot reaction both (−)-*trans*-CBDs and (−)-*trans*-Δ^9^-THCs, avoiding cumbersome and longer procedure to selectively prepare the (−)-*trans*-Δ^9^-THCs, without the awkward formation of their Δ^8^ isomers. The synthesis of the monomethyl ether derivatives of CBD, THC and CBG is reported in Fig. 3A, Scheme 2. (−)-*trans*-CBDM and CBGM were easily prepared by methylation of the commercially available CBD and CBG by reaction with 0.5 equivalents of dimethylsulfate, in DMF at room temperature, using K_2_CO_3_ as base (Fig. 3A, Scheme 3). In contrast (−)-*trans*-Δ^9^-THCM was prepared from (−)-*trans*-CBDM, through cyclization catalyzed by pTSA (Fig. 3A, Scheme 2). The chemical identification of synthetic (−)-*trans*-CBDH, (−)-*trans*-Δ^9^-THCH, (−)-*trans*-CBDM, (−)-*trans*-Δ^9^-THCM and CBGM, and their unambiguous ^1^H and ^13^C assignments were achieved by NMR spectroscopy (Figure SI-1–5, Supporting Information). In particular for (−)-*trans*-CBDH and (−)-*trans*-Δ^9^-THCH, as already stated during the synthesis of (−)-*trans*-CBDB, (−)-*trans*-CBDP, (−)-*trans*-Δ^9^-THCB and (−)-*trans*-Δ^9^-THCP, and by comparison with the well-known homologs (CBD, CBDV, Δ^9^-THC, and Δ^9^-THCV) no significant differences in the proton and carbon chemical shifts of the terpene and aromatic moieties were observed among CBD and Δ^9^-THC homologs. The sole exception observed regards the integration of the multiplet in the range 1.4–1.2 ppm in the ^1^H spectra and the number of carbon signals in the range 20–30 ppm of the ^13^C spectra, corresponding to the central methylene units of the alkyl chain on the resorcinyl moiety. The perfect match in the chemical shift of the terpene and aromatic moieties between the synthesized (−)-*trans*-CBDH and (−)-*trans*-Δ^9^-THCH and the respective homologues^2–4,8^, combined with the mass spectra and fragmentation pattern, allowed us to unambiguously confirm the chemical structures of the two new synthetic cannabinoids.The trans (1R,6R) configuration at the terpene moiety was confirmed by optical rotatory power. The new cannabinoids (−)-*trans*-CBDH, (−)-*trans*-Δ^9^-THCH, (−)-*trans*-CBDM and (−)-*trans*-Δ^9^-THCM showed an [α]_D_^20^ of − 146°, − 166°, − 113° and − 161°, respectively, in chloroform. The [α]_D_^20^ values were in line with those of the homologs^2,8,15^, suggesting a (1R,6R) configuration for the four phytocannabinoids. Lastly, the perfect superimposition between the ^1^H and ^13^C NMR spectra of both synthetic and extracted (−)-*trans*-CBDH was observed, confirming the identity of the new cannabinoids identified in the FM2 cannabis variety (Figure SI-6, Supporting Information).

A comparison of the retention time, molecular ion and fragmentation spectra of each pure synthesized standard with those found in FM2 led us to conclude that the first peak A_d_ could be assigned to CBDH and the second one B_d_ to CBDM (Fig. 3B). The third peak C_d_ could most likely be associated to Δ^9^-THCH although its very low abundance and the presence of other interferents in the fragmentation spectrum from the FM2 extract did not allow an unambiguous assignment of its chemical structure (Fig. 3B). Moreover, no trace of Δ^9^-THCM was found. Fragmentation in negative ionization mode helped us to distinguish between CBDH and Δ^9^-THCH, which were identical in positive ionization mode, whereas no ionization was obtained in negative mode for Δ^9^-THCM due to the lack of free hydroxyl groups to be deprotonated (Fig. 3B). Confirmation of the identification of CBDH was achieved by isolation of pure fractions from the FM2 extract containing the acidic precursor CBDHA by semipreparative liquid chromatography. The pure compound was decarboxylated by heat and analysed by UHPLC-HESI-Orbitrap. Unfortunately, it was not possible to isolate fractions of FM2 containing THCHA due to its very low abundance. However, the stereoselective synthesis of Δ^9^-THCH allowed to assign a certain chemical structure to the corresponding peak in the FM2 sample.

### Semi-quantification of CBDH and Δ^9^-THCH in the FM2 extract

Thanks to the synthetically prepared analytical standards of CBDH, Δ^9^-THCH, CBDM and CBGM, we were able to provide a semi-quantification of these cannabinoids in the FM2 cannabis variety by building the corresponding calibration curves. The results of concentration were in the order of the µg/g, while the main cannabinoids CBD and Δ^9^-THC were in the order of the mg/g (56 and 39 mg/g respectively). In particular, the hexyl homologs of CBD and Δ^9^-THC resulted 27 µg/g and 7 µg/g, while the methyl ether derivatives CBDM and CBGM were 50 µg/g and 102 µg/g. No Δ^9^-THCM was detected in the FM2.

### Effects of CBDH on the formalin test in mice

Formalin paw injection is a solid and widely used model of nociception with high face validity when tested with analgesic drugs. A nociceptive response to subcutaneous formalin induced an early, short-lasting first phase (0–7 min) followed by a quiescent period, and then a second, prolonged phase (15–60 min) of tonic hyperalgesia (Fig. 3C). In the tonic phase, two-way ANOVA revealed that CBDH (1, 2 mg/kg, i.p.) significantly reduced the late phase of the formalin-induced nocifensive behavior when compared to the vehicle-treated group (treatment F_(4,288)_ = 17.32, *P* < 0.0001, time F_(12,288 )_ = 67.80, *P* < 0.0001 and interaction F_(48,288)_ = 3.02, *P* < 0.0001); also, the dose of 2 mg/kg had a significant antinociceptive effect as compared to the vehicle group. The doses of 3 and 5 mg/kg had no effect on the formalin test (Fig. 3C).

## Discussion

The comprehensive characterization of the chemical profile of a cannabis variety is a rather arduous task as the analytical tools in the chemist’s hand are not able to cover such a broad range of compounds. However, the high sensitivity and selectivity of the high-resolution mass spectrometry, for example those achieved with the Orbitrap technology, can enable the identification of a reasonable number of molecules, even when present in very small traces. This approach allowed for the identification of new series of cannabinoids, CBD and THC homologs, with different lengths of the alkyl side chain, which were recently reported by our group^2–4,8^. The present work expanded the scope of cannabinoids identification completing the series of homologs with different alkyl side chain from three to seven methylene units. Up to now, only cannabinoids with an odd number of carbon atoms on the side chain have been reported and those with an even number of carbon atoms have been supposed to be artifacts derived from fungal ω-oxidation of their corresponding homologs^7^. The investigation of the origin of these species, such as those with a butyl and hexyl side chain, is beyond the scope of this work, but, although surprising, it is certain that such cannabinoids are actually present in a medicinal cannabis variety. The literature reports the existence of monomethyl ether derivatives of the canonical pentyl cannabinoids to justify the presence of compounds bearing an additional methyl group. Although on one side the structural identity of such derivatives was confirmed, our findings pointed out a new series of cannabinoids with the same molecular formula of the monomethyl ethers but with a different arrangement. Their origin, whether it is from the plant or from microorganisms, should be investigated as this might disclose new insights in the cannabis biochemistry. It is certainly important to underline that it is very easy to confuse CBDH and CBDM, as well as Δ^9^-THCH and Δ^9^-THCM. However, the match of the high-resolution fragmentation patterns with their pure synthetic standards was determinant to assign the respective chemical structure. This work might serve in the future for any researcher to distinguish between two species that show a very similar analytical behaviour. In a similar way, the methyl ether derivative of CBG was also identified (CBGM).

It is worth noting that no Δ^9^-THCM was detected in the FM2 variety. On the other hand, both CBDM and CBGM showed a high peak as well as their native precursors CBDMA and CBGMA. Achieved results are in accordance with what reported by Lumır Ondrej Hanus et al., which showed that the cannabigerol monomethyl ether is always presents in greater quantities than its products, THCMA and CBDMA^7,16^.

de Meijer et al. demonstrated that the cannabinoid acid synthases (THCAS, CBDAS) show a different affinity for CBGA alkyl homologues. This concept would explain the achieved results. CBDAS could be competitively stronger than THCAS when the substrate is CBGMA^17,18^.

The comparison of the results obtained for the concentrations of unorthodox cannabinoids in the FM2 variety suggested that there is no relationship between the different series as the same CBD to THC ratio is not respected. Considering our recent work on the heptyl derivatives of CBD and Δ^9^-THC, CBDP and Δ^9^-THCP were found in the FM2 at the concentration of 243 µg/g and 29 µg/g^4^; whereas, in the same cannabis variety, the butyl series of CBD and THC was found at the concentration of 500 µg/g and 400 µg/g for CBDB and Δ^9^-THCB respectively^8^. In this work, we found 27 µg/g and 7 µg/g for CBDH and Δ^9^-THCH respectively. However, this data should be verified considering a larger number of different varieties in order to provide a reliable statistical significance.

The CBDH could have pleiotropic mechanisms of action through which it can exert its pharmacological effect. We found that the doses of the 1 and 2 mg/Kg significantly reduced the late phase of the formalin-induced nocifensive behavior, whereas the higher doses 3 and 5 mg/Kg were uneffective. This could be due, at least in part, assuming that at these doses CBDH can activate receptor facilitating nociception such as TRPV1 or other channels. On the other hand, we can speculate that CBDH at the higher doses could block receptors involved in antinociception such as CB1 or CB2. However, further pharmacological studies are needed to better investigate the pharmacodynamics profile of this interesting compound.

Another piece of knowledge towards understanding *Cannabis Sativa* L. cannabinoma has been added with this work. In particular, clarity has been made about the possible confusion between phytocannabinoids with a 6-term alkyl chain (CBDH, THCH) and those with a methylated resorcinolic hydroxyl group (CBDM, THCM). Furthermore, two new phytocannabinoids CBDH and THCH have been identified in the FM2 variety by comparison with their respective authentic synthesized compounds. In particular, CBDH has been isolated and its pharmacological activity has been evaluated in vivo in mice. At extremely low doses (1 mg/kg) it showed an interesting nocifensive activity. However, the CBDH concentration of 27 μg/g found in the FM2 variety is too low to exert the pharmacological effect but it is not excluded that other cannabis varieties may contain higher concentrations. More in-depth pharmacological studies are currently underway to clarify the mechanism of action of this new phytocannabinoid.

## Methods

### Plant material

FM2 cannabis variety is produced from the strain CIN-RO bred by the Council for Agricultural Research and Economics (CREA) in Rovigo (Italy) and supplied to the Military Chemical Pharmaceutical Institute (MCPI, Firenze, Italy). Experiments on FM2 inflorescence (batch n. 6A32/1) were performed with the authorization of the Italian Ministry of Health (prot. n. SP/062). Two 5 g packs were finely grinded (< 2 mm particle size) and divided into two batches: 500 mg were extracted with 50 mL of ethanol 96% according to the procedure reported in the monograph of Cannabis Flos of the German Pharmacopoeia^19^ and analyzed by UHPLC-HESI-Orbitrap without further dilution. The remaining 9.5 g were treated according to the protocol of Pellati et al. with minor changes^20^. Briefly, freeze-dried plant material was sonicated with 400 mL of *n*-hexane for 15 min in an ice bath. After centrifugation for 10 min at 2000 × *g* the pellets were discarded. The same procedure was repeated twice more. The supernatants were then dried under reduced pressure and resuspended in 10 mL of acetonitrile, filtered and passed through a semi-preparative liquid chromatography for the isolation of the acidic species of the cannabinoids of interest.

### Isolation of natural CBDH

A semi-preparative LC system (Octave 10 Semba Bioscience, Madison, USA) was used to separate the FM2 mixture into 80 fractions in a total run time of 80 min. The chromatographic conditions used are reported in the paper by Citti et al.^4^. A Luna C_18_ with a fully porous silica stationary phase (Luna 5 µm C18(2) 100 Å, 250 × 10 mm) (Phenomenex, Bologna, Italy) was the column employed and a mixture of acetronitrile:0.1% aqueous formic acid 70:30 (v/v) was used as mobile phase at a constant flow rate of 5 mL/min. The fractions containing CBDHA (retention time 13.0 min) was isolated as reported in our previous work^2^. The fractions containing CBDHA (13.0 min) were analyzed by UHPLC-HESI-Orbitrap and dried on the rotavapor at 70 °C. The residue was placed in an oven at 120 °C for 2 h to achieve decarboxylation. An amount of about 0.3 mg of CBDH was obtained.

### UHPLC-HESI-Orbitrap metabolomic analysis

Analyses on FM2 extracts were performed on a Thermo Fisher Scientific Ultimate 3000 provided with a vacuum degasser, a binary pump, a thermostated autosampler, a thermostated column compartment and interfaced to a heated electrospray ionization source and a Q-Exactive Orbitrap mass spectrometer (UHPLC-HESI-Orbitrap). The HESI and Orbitrap parameters were set following our previous work^4^. Briefly, the capillary temperature was set at 320 °C, the vaporizer temperature at 280 °C, the electrospray voltage at 4.2 kV (for the positive ionization mode) and 3.8 kV (for the negative mode), the sheath gas and the auxiliary gas at 55 and 30 arbitrary units respectively, the RF level of the S lens at 45. Analyses were acquired in full scan data-dependent acquisition (FS-dd-MS^2^) in positive and negative mode with a resolving power of 70,000 FWHM and *m*/*z* of 200 using the Xcalibur 3.0 software (Thermo Fisher Scientific, San Jose, CA, USA). For the Orbitrap mass analyzer, a scan range of *m*/*z* 250–400, an AGC of 3e6, an injection time of 100 ms and an isolation window of *m*/*z* 0.7 were chosen as the optimal parameters. The collision energy for the fragmentation of the molecular ions was set at 20 eV. The exact masses of the [M+H]^+^ and [M−H]^−^ molecular ions were extracted from the total ion chromatogram (TIC) of the FM2 extracts and matched with pure analytical standards for accuracy of the exact mass (5 ppm), retention time and MS/MS spectrum.

The chromatographic separation was carried out on a core shell C_18_ stationary phase (Poroshell 120 SB-C18, 3.0 × 100 mm, 2.7 µm, Agilent, Milan, Italy) following the conditions employed for our previous work^4^.

A semi-quantitative analysis of Δ^9^-THC and CBD, their hexyl homologs CBDH and Δ^9^-THCH, and the methyl ether derivatives of CBD and CBG, CBDM and CBGM, was carried out using a calibration curve with the external standard method. A stock solution of CBD and Δ^9^-THC (1 mg/mL) was properly diluted to obtain five non-zero calibration points at the final concentrations of 50, 100, 250, 500 and 1000 ng/mL; a stock solution of CBDH, CBDM, Δ^9^-THCH, CBDM and CBGM was diluted to obtain the final concentrations of 5, 25, 50, 100 and 250 ng/mL. The linearity was assessed by the coefficient of determination (*R*^2^), which was greater than 0.992 for each analyte.

### Synthetic procedure

The reagents and the solvents used for the synthesis of the analytical standards were purchased from Sigma-Aldrich and VWR, respectively. In the synthetic procedures, the solvents were abbreviated as following: acetonitrile (ACN); chloroform (CHCl_3_); cyclohexane (CE); dichloromethane (DCM); diethyl ether (Et_2_O); dimethyl sulfoxide (DMSO); ethyl acetate (AcOEt). The reactions were monitored by thin-layer chromatography (TLC) using 60F-254 silica gel plates (from Merck) and inspected with UV lamp, or alkaline KMnO_4_ stain. Purification of the synthesized products was performed by flash chromatography on silica gel (40–63 μm). The mobile phase is specified in the respective following monographies. NMR spectra were recorded on a Bruker 400 (at 400.134 MHz for ^1^H and 100.62 MHz for ^13^C) or on a Bruker 600 (at 600.130 MHz for ^1^H and 150.902 MHz for ^13^C) spectrometer. Chemical shifts (δ) are reported in parts per million (ppm) and referenced to the solvent residual peaks. Coupling constants are reported in hertz (Hz) and the splitting pattern is reported as: singlet (s), doublet (d), triplet (t), quartet (q), double doublet (dd), quintet (quin), multiplet (m), broad signal (b). Monodimensional and bidimensional spectra were acquired using the same parameters previously reported^3–5,8^. Optical rotation (α) was acquired with a Polarimeter 240C from Perkin–Elmer (Milan, Italy), using a cell with a length of 100 mm, and a volume 1 mL.

#### *Synthesis of (3,5-dimethoxybenzyl)triphenylphosphonium bromide (1)*

Triphenylphosphine (6.3 g, 23.8 mmol, 1.1 equiv.) was added to a stirred solution of 1-(bromomethyl)-3,5-dimethoxybenzene (5.0 g, 21.6 mmol, 1 equiv.), in 30 mL of toluene and refluxed for 6 h. After standing at room temperature overnight, the precipitate formed was collected by filtration, washed with diethyl ether and dried to give 10.4 g of a white solid (quant. yield).

^1^H-NMR (400 MHz, CDCl_3_) δ 7.78–7.76 (m, 9H), 7.66–7.63 (m, 6H), 6.35 (t, 2H, *J* = 2.3 Hz), 6.30 (q, 1H, *J* = 2.3 Hz), 5.32 (d, 2H, *J* = 14.3 Hz), 3.54 (s, 6H).

#### *Synthesis of (E*/*Z)-1-(hexyl-1-en-1-yl)-3,5-dimethoxybenzene (2)*

Valeraldehyde (0.48 mL, 4.56 mmol, 1.5 equiv.) was added to a stirred suspension of **1** (1.5 g, 3.04 mmol, 1 equiv.) in 20 mL of aqueous 0.1 M K_2_CO_3_. The mixture was refluxed overnight and chilled down at 0 °C. Cyclohexane (20 mL) was added, and the biphasic mixture was vigorously stirred in the same condition for two hours in order to precipitate triphenylphosphine oxide. The solid was removed by filtration and the organic phase separated. The aqueous layer was extracted two more times with cyclohexane. The combined organic phase was washed with brine, dried over anhydrous Na_2_SO_4_ and concentrated to give 540 mg (81% yield) of a yellow oil. The product was obtained as a 55:45 E/Z mixture of alkene, pure enough to be used in the next step without further purification.

^1^H NMR (400 MHz, CDCl_3_, Z-isomer) δ 6.43 (d, 2H, *J* = 2.2 Hz), 6.35 (t, 1H, *J* = 2.2 Hz), 6.31 (d, 1H, *J* = 11.5 Hz), 5.66 (dt, 1H, *J* = 11.5, 7.0 Hz), 3.79 (s, 6H), 2.37–2.30 (m, 2H), 1.47–1.31 (m, 4H), 0.90 (t, 3H, *J* = 7.1 Hz); ^1^H NMR (400 MHz, CDCl_3_, E-isomer) δ 6.51 (d, 2H, *J* = 2.2 Hz), 6.29 (bm, 1H), 6.21 (dt, 1H, *J* = 15.9, 6.85 Hz), 3.79 (s, 6H), 2.24–2.18 (m, 2H), 1.47–1.31 (m, 4H, overlap with the same signals of Z-isomer), 0.92 (t, 3H, *J* = 7.1 Hz).

#### *Synthesis of 1-hexyl-3,5-dimethoxybenzene (3)*

The mixture of (E/Z)-1-(hept-1-en-1-yl)-3,5-dimethoxybenzene (**2**), solubilized in EtOH, was selectively reduced at the double bond by hydrogenation with the flux reactor H-Cube Mini Plus ThalesNano using the following conditions: temperature 30 °C, H_2_ 20 psi, cartridge Pd/C, solvent EtOH, flow 1 mL/min. The solvent was evaporated obtaining 495 mg (91% yield) of a colourless liquid pure enough to be used in the next step without further purification.

^1^H NMR (400 MHz, CDCl_3_) δ 6.37 (d, *J* = 2.3 Hz, 2H), 6.32 (t, *J* = 2.3 Hz, 1H), 3.80 (s, 6H), 2.57 (t, *J* = 7.48 Hz, 2H), 1.61 (qnt, 2H, *J* = 6.9 Hz), 1.39–1.28 (m, 6H), 0.91 (t, *J* = 6.4 Hz, 3H). ^13^C NMR (101 MHz, CDCl_3_) δ 160.67, 145.43, 106.48, 97.55, 55.23, 36.32, 31.73, 31.25, 29.02, 22.61, 14.10.

#### *Synthesis of 5-hexylbenzene-1,3-diol (4)*

**3** (495 mg, 2.23 mmol, 1 equiv.) was solubilized in anhydrous DCM at − 15 °C and under argon atmosphere, and a 1 M solution of BBr_3_ in anhydrous DCM (5 mL, 4.9 mmol, 2.2 equiv.) was added dropwise over a period of 30 min. The mixture was stirred at room temperature overnight and quenched with an aqueous saturated solution of NaHCO_3_. The organic phase was washed with water, brine, dried over anhydrous Na_2_SO_4_ and concentrated to give 430 mg (99% yield) of an orange liquid which crystalized upon standing.

^1^H NMR (400 MHz, CDCl_3_) δ 6.24 (d, 2H, *J* = 2.3 Hz), 6.17 (t, 1H, *J* = 2.3 Hz), 4.71 (bs, 2H), 2.49 (t, 2H, *J* = 8.0 Hz), 1.57 (qnt, 2H, *J* = 8.0 Hz), 1.35–1.23 (bm, 6H), 0.88 (t, 3H, *J* = 6.8 Hz). ^13^C NMR (101 MHz, CDCl_3_) δ 156.57, 146.16, 108.04, 100.12, 35.82, 31.74, 31.01, 28.94, 22.59, 14.09.

#### *Synthesis of (1′R,2′R)-4-hexyl-5′-methyl-2′-(prop-1-en-2-yl)-1′,2′,3′,4′-tetrahydro-[1,1′-biphenyl]-2,6-diol, (−)-trans-CBDH and (6aR,10aR)-3-hexyl-6,6,9-trimethyl-6a,7,8,10a-tetrahydro-6H-benzo[c]chromen-1-ol, (−)-trans-Δ*^9^*-THCH*

(1*S*,4*R*)-1-methyl-4-(prop-1-en-2-yl)cycloex-2-enol (304 mg, 2.0 mmol, 0.9 eq.), solubilized in 20 mL of anhydrous DCM, was added dropwise over a period of 20 min to a stirred solution of 5-hexylbenzene-1,3-diol (**1**) (433 mg, 2.23 mmol, 1 eq.) and *p*-toluenesulfonic acid (40 mg, 0.2 mmol, 0.1 eq.) in anhydrous DCM (20 mL) at room temperature and under argon atmosphere. The reaction was stirred in the same conditions and monitored every 15 min by HPLC, following the same chromatographic method using for the analytic characterization. After for 2 h, the putative ratio between CBDH and THCH was almost 1:1 and no traces of *Δ*^*8*^*-THCH* were detected. The reaction was therefore quenched with 20 mL of a saturated aqueous solution of NaHCO_3_. The organic layer was washed with brine, dried over anhydrous Na_2_SO_4_ and evaporated. The crude was chromatographed over silica gel (ratio crude:silica 1/150, eluent: CE:DCM 8/2). All the chromatographic fractions were analyzed by HPLC–UV and UHPLC-HESI-Orbitrap and only the fractions containing exclusively CBDH and THCH were separately collected to give 65 mg of CBDH as colorless oil (10% yield, purity > 99%) and 71 mg of THCH as a light purple oil (11% yield, purity > 99%). These two fractions were used as pure analytic standards for spectroscopic and analytic characterization. The chromatographic fraction containing both CBDH and THCH (c.a. 150 mg) was purified by semipreparative HPLC on a C18 reverse phase using ACN:water 70:30 as mobile phase. Two other aliquots of CBDH (45 mg) and THCH (60 mg) were obtained.

(−)-*trans*-CBDH: colorless oil. 110 mg (17% yield). ^1^H NMR (400 MHz, CDCl_3_) δ 6.10–6.32 (bm, 2H), 5.97 (bs, 1H), 5.57 (s, 1H), 4.73–4.59 (bm, 2H), 4.56 (s, 1H), 3.88–3.81 (m, 1H), 2.46–2.36 (m, 3H), 2.27–2.20 (m, 1H), 2.09 (ddt, *J* = 2.4, 5.0, 18.0 Hz, 1H), 1.85–1.76 (m, 5H), 1.65 (s, 3H), 1.58–1.51 (m, 2H), 1.31–1.25 (m, 6H), 0.87 (t, *J* = 6.7 Hz, 3H). ^13^C NMR (101 MHz, CDCl_3_) δ 156.14, 153.98, 149.54, 143.20, 140.19, 124.26, 113.89, 110.97, 109.78, 108.12, 46.29, 37.42, 35.65, 31.86, 31.05, 30.54, 29.09, 28.54, 23.82, 22.73, 20.67, 14.22. HRMS m/z [M+H]^+^ calcd. for C_22_H_33_O_2_^+^: 329.2475. Found: 343.2629; [M−H]^−^ calcd. for C_22_H_31_O_2_^−^: 327.2330. Found: 341.2482. [α]_D_^20^ = −146° (c = 1.0, ACN).

(−)-*trans*-Δ^9^-THCH: light purple oil. 131 mg (20% yield). ^1^H NMR (400 MHz, CDCl_3_) δ 6.30 (bt, 1H), 6.27 (bd, 1H), 6.14 (bd, 1H), 4.78 (s, 1H), 3.20 (dt, *J* = 2.5, 10.8 Hz, 1H), 2.43 (t, *J* = 7.5 Hz, 2H), 2.16–2.18 (m, 2H), 1.95–1.88 (m, 1H), 1.71–1.65 (m, 4H), 1.58–1.51 (m, 2H), 1.43–1.36 (m, 4H), 1.34–1.24 (m, 6H), 1.09 (s, 3H), 0.88 (t, *J* = 6.8 Hz, 3H). ^13^C NMR (101 MHz, CDCl_3_) δ 154.91, 154.29, 142.97, 134.54, 123.87, 110.24, 109.17, 107.69, 77.35, 45.95, 35.66, 33.72, 31.88, 31.31, 31.08, 29.16, 27.71, 25.16, 23.51, 22.73, 19.41, 14.25. HRMS m/z [M+H]^+^ calcd. for C_22_H_33_O_2_^+^: 329.2475. Found: 343.2629; [M−H]^−^ calcd. for C_22_H_31_O_2_^−^: 327.2330. Found: 341.2482. [α]_D_^20^ = −166° (*c* 1.0, ACN).

#### *Synthesis of (1′R,2′R)-6-methoxy-5′-methyl-4-pentyl-2′-(prop-1-en-2-yl)-1′,2′,3′,4′-tetrahydro-[1,1′-biphenyl]-2-ol, *(−)*-trans-CBDM*

To a solution of (−)*-trans-CBD* (500 mg, 1.6 mmol, 1 equiv.) in dry DMF (5 mL), K_2_CO_3_ (414 mg, 3.2 mmol, 2 equiv.) and dimethylsulphate (76 μL, 0.8 mmol, 0.5 equiv.) were added and stirred at room temperature overnight. The mixture was quenched with water and extracted with Et_2_O. The organic phase was washed with brine, dried over anhydrous Na_2_SO_4_ and concentrated. The titled compound was purified by column chromatography (eluent CE:AcOEt 95:5) to give 162 mg (31% yield) of an amber liquid.

^1^H NMR (400 MHz, CDCl_3_) δ 6.30 (s, 1H), 6.22 (s, 1H), 5.99 (bs, 1H), 5.57 (bs, 1H), 4.49 (s, 1H), 4.32 (s, 1H), 3.99 (bd, 1H), 3.70 (s, 3H), 2.49 (t, *J* = 7.6 Hz, 2H), 2.43–2.37 (m, 1H), 2.25–2.19 (m, 1H), 2.07 (bdt, 1H), 1.80–1.74 (m, 6H),1.65 (s, 3H), 1.62–1.55 (m, 3H), 1.35–1.26 (m, 4H), 0.87 (t, *J* = 6.8 Hz, 3H). ^13^C NMR (101 MHz, CDCl_3_) δ 158.31, 155.88, 147.41, 142.82, 139.70, 124.68, 115.20, 111.02, 109.67, 103.27, 55.70, 46.81, 36.16, 35.65, 31.71, 30.97, 30.51, 28.26, 23.84, 22.70, 18.87, 14.19. HRMS m/z [M+H]^+^ calcd. for C_22_H_33_O_2_^+^: 329.2475. Found: 343.2629; [M−H]^−^ calcd. for C_22_H_31_O_2_^−^: 327.2330. Found: 341.2482. [α]_D_^20^ = −113° (*c* 1.0, ACN).

#### *Synthesis of (E)-2-(3,7-dimethylocta-2,6-dien-1-yl)-3-methoxy-5-pentylphenol (CBGM)*

The title compound was synthesized and purified according to the procedure described for (−)*-trans-CBDM*.

Yellow liquid (57% yield). ^1^H NMR (400 MHz, CDCl_3_) δ 6.33 (s, 1H), 6.31 (s, 1H), 5.24 (dt, *J* = 7.6, 1.6 Hz, 1H), 5.20 (s, 1H), 5.05 (dt, *J* = 7.6, 1.6 Hz, 1H), 3.80 (s, 3H), 3.38 (d, *J* = 7.2 Hz, 2H), 2.51 (t, *J* = 7.2 Hz, 2H), 2.10–2.03 (m, 4H), 1.79 (s, 3H), 1.67 (s, 3H), 1.64–1.57 (m, 5H), 1.34–1.29 (m, 4H), 0.89 (t, *J* = 6.8 Hz, 3H). ^13^C NMR (101 MHz, CDCl_3_) δ 157.84, 155.54, 142.67, 138.09, 131.98, 124.08, 122.34, 112.42, 109.04, 103.71, 55.62, 39.87, 36.21, 31.73, 31.14, 26.63, 25.80, 22.71, 22.21, 17.83, 16.28, 14.18. HRMS m/z [M+H]^+^ calcd. for C_22_H_35_O_2_^+^: 331.2632. Found: 343.2629; [M−H]^−^ calcd. for C_22_H_33_O_2_^−^: 329.2486. Found: 341.2482.

#### *Synthesis of (6aR,10aR)-1-methoxy-6,6,9-trimethyl-3-pentyl-6a,7,8,10a-tetrahydro-6H-benzo[c]chromene, *(−)*-trans-THCM*

To a solution of (−)*-trans-CBDM* (100 mg, 0.32 mmol, 1 equiv.) in dry DCM (10 mL), at room temperature and under nitrogen atmosphere, *p*TSA (5 mg, 0.03 mmol, 0.1 equiv.) was added. The solution was stirred in the same condition, monitoring the progress of the reaction by HPLC–UV/Vis in order to avoid the further conversion of the forming (−)*-trans-Δ*^*9*^*-THCM* into the Δ^8^ isomer. After 1 h, the conversion is completed, and the reaction was quenched with NaHCO_3_ aqueous. The organic phase was washed with brine, dried over anhydrous Na_2_SO_4_ and concentrated. The crude was purified by column chromatography (eluent CE:DCM 9:1) to give 45 mg (43% yield) of colorless liquid.

^1^H NMR (400 MHz, CDCl_3_) δ 6.30 (s, 1H), 6.26 (s, 1H), 6.23 (s, 1H), 3.84 (s, 3H), 3.17 (dt, *J* = 11.8, 2.0 Hz, 1H), 2.50 (t, *J* = 7.6 Hz, 2H), 2.16–2.14 (m, 2H), 1.93–1.88 (m, 1H), 1.70–1.57 (m, 7H), 1.43–1.30 (m, 11H), 1.08 (s, 3H), 0.89 (t, *J* = 6.4 Hz, 3H). ^13^C NMR (101 MHz, CDCl_3_) δ 158.52, 154.50 142.70, 133.59, 124.98, 110.52, 110.37, 103.07, 55.31, 46.05, 36.19, 34.06, 31.76, 31.44, 30.98, 27.72, 27.07, 25.30, 23.55, 22.71, 19.29, 14.18. HRMS m/z [M+H]^+^ calcd. for C_22_H_33_O_2_^+^: 329.2475. Found: 343.2629; [M−H]^−^ calcd. for C_22_H_31_O_2_^−^: 327.2330. Found: 341.2482. [α]_D_^20^ = −161° (*c* 1.0, ACN).

### Formalin test in mice

The formalin test assay was performed as previously reported in Linciano et al.^8^. In detail, male C57BL/6J mice, 6–8 weeks (Envigo, Italy), were housed under controlled conditions (12 h light/12 h dark cycle; temperature 20–22 °C; humidity 55–60%) with chow and tap water available ad libitum. All surgeries and experimental procedures were approved by the Animal Ethics Committee of the University of Campania “L. Vanvitelli”, Naples (prot. no. 1066/2016 PR). Animal care was in compliance with Italian (D.L. 116/92) and European Commission (O.J. of E.C. L358/1 18/12/86) regulations on the protection of laboratory animals. Efforts were made to minimize animal suffering and to reduce the number of animals used. All experiments were performed in a randomized manner by the same operator blind to pharmacological treatments. Mice were used after a 1-week acclimation period and received formalin (1.25% in saline, 30 μL) in the dorsal surface of one side of the hind paw. Each mouse, randomly assigned to one of the experimental groups (n = 5–6), was placed in a plexiglass cage and allowed to move freely for 15–20 min. A mirror was placed at a 45° angle under the cage to allow full view of the hind paws. Lifting, favoring, licking, shaking, and flinching of the injected paw were recorded as nocifensive behavior. The total time of the nociceptive response was measured every 5 min for 1 h and expressed in minutes (mean ± SEM). Mice received vehicle (0.5% DMSO in saline) or different doses of CBDH (1,2, 3, and 5 mg/kg, i.p.) 20 min before formalin injection^8^.

## Supplementary Information

Supplementary Information.
