# Supplementary material for: Identification of a new cannabidiol n-hexyl homolog in a medicinal cannabis variety with an antinociceptive activity in mice: cannabidihexol
Source: Sci Rep. 2020 Dec 16;10:22019. doi: 10.1038/s41598-020-79042-2 (PMC7744557; doi:10.1038/s41598-020-79042-2)
Supplement: Supplementary file 1 — Supplementary Information. [file 41598_2020_79042_MOESM1_ESM.docx]

**Identification of a new cannabidiol n-hexyl homolog in a medicinal cannabis variety with an antinociceptive activity in mice: cannabidihexol.**

Pasquale Linciano^1,†^, Cinzia Citti^1,2,3,†^, Fabiana Russo^1^, Francesco Tolomeo^2^, Aldo Laganà^3,4^, Anna Laura Capriotti^4^, Livio Luongo^5^, Monica Iannotta^5^, Carmela Belardo^5^, Sabatino Maione^5^, Flavio Forni^1^, Maria Angela Vandelli^1^, Giuseppe Gigli^3^, Giuseppe Cannazza^1,3,*^

1. Department of Life Sciences, University of Modena and Reggio Emilia, Via Campi 103, 41125 Modena, Italy
2. Mediteknology (CNR spin-off company), Via Arnesano, 73100 Lecce
3. CNR NANOTEC, Istituto di Nanotecnologia, Via Monteroni, 73100 Lecce, Italy
4. Department of Chemistry, Sapienza University of Rome, Piazzale Aldo Moro 5, 00185 Rome, Italy
5. Division of Pharmacology, Università degli studi della Campania "L. Vanvitelli", Via Costantinopoli, 16, 80138, Naples, Italy

^†^ These authors contributed equally to the work

- Corresponding author: [giuseppe.cannazza@unimore.it](mailto:giuseppe.cannazza@unimore.it), tel: +39 059 2055013, fax: +39 059 2055750, ORCID ID: 0000-0002-7347-7315

**Table of Content**

| **Figure SI-1.** NMR spectroscopic characterization of synthetic (-)-*trans*-CBDH | … SI-2 |
| --- | --- |
| **Figure SI-2.** NMR spectroscopic characterization of synthetic (-)-*trans*-Δ^9^-THCH | … SI-5 |
| **Figure SI-3.** NMR spectroscopic characterization of synthetic (-)-*trans*-CBDM | … SI-8 |
| **Figure SI-4.** NMR spectroscopic characterization of synthetic (-)-*trans*-Δ^9^-THCM | … SI-9 |
| **Figure SI-5.** NMR spectroscopic characterization of synthetic CBGM | …SI-10 |
| **Figure SI-6.** Superimpostion of 1H-NMR and 13C-NMR spectra of synthetic and extracted (-)-trans-CBDH | …SI-11 |
|  |  |

| **Figure SI-1.** NMR spectroscopic characterization of synthetic (-)-*trans*-CBDH |
| --- |
| 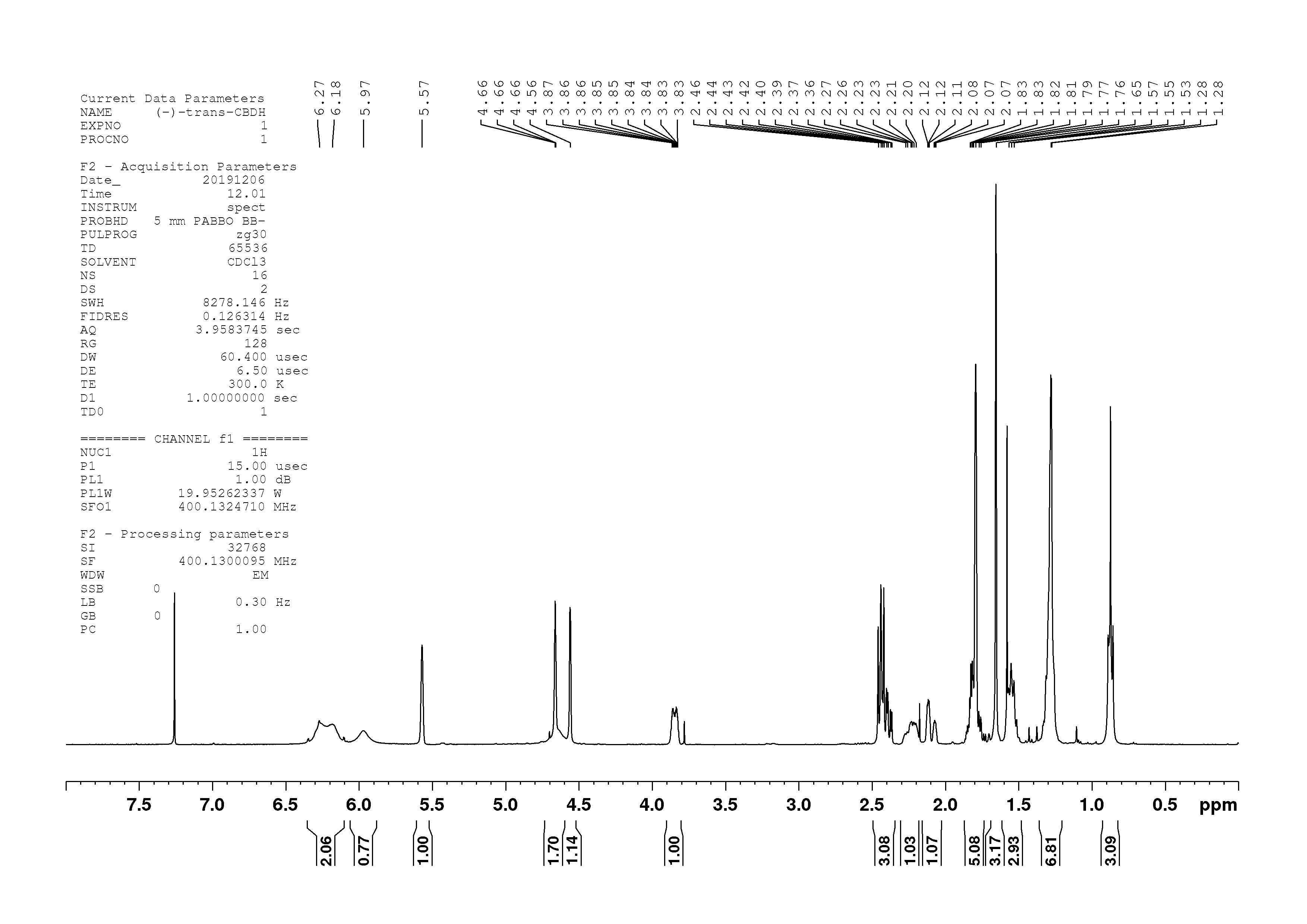 |
| 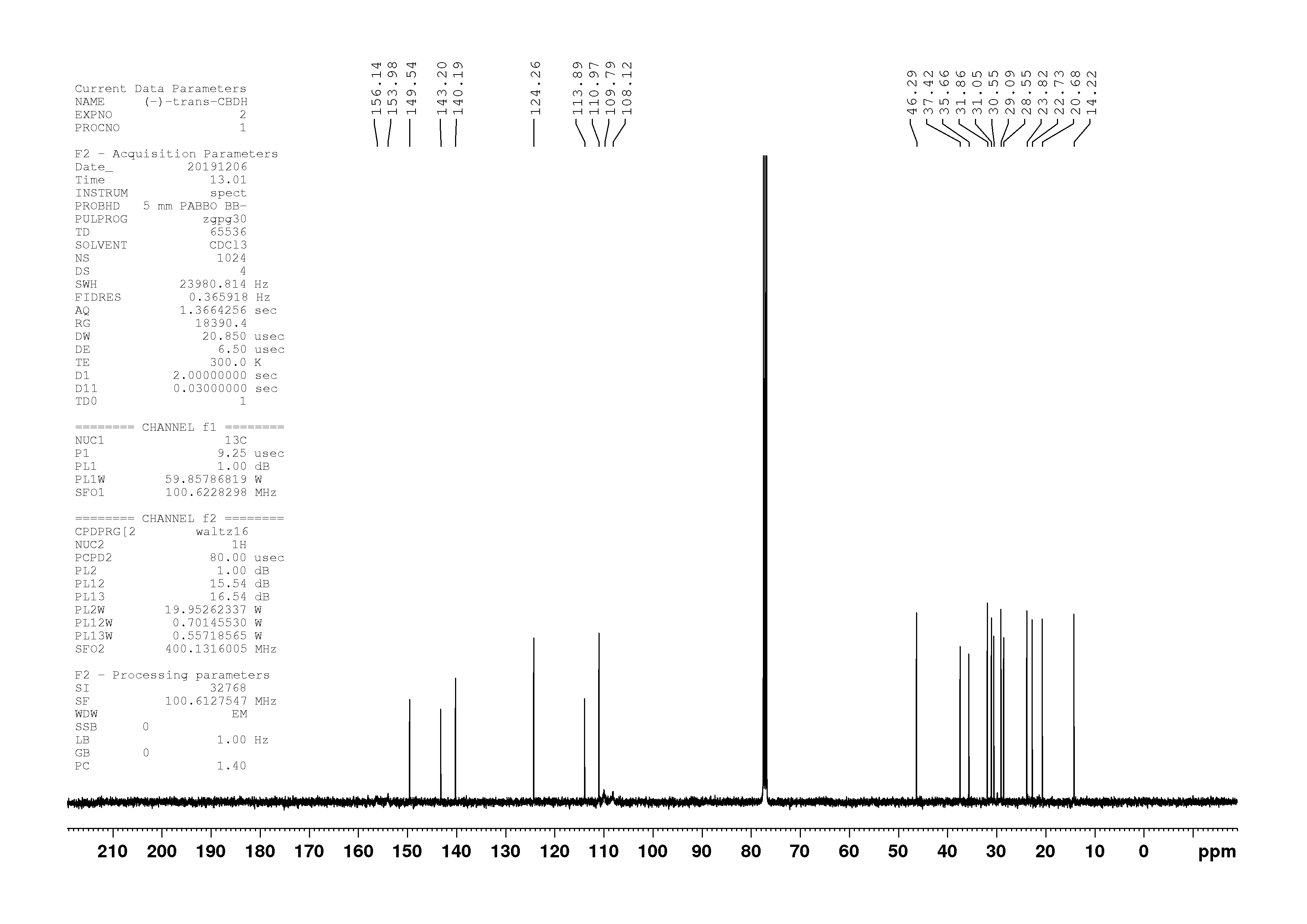 |
| 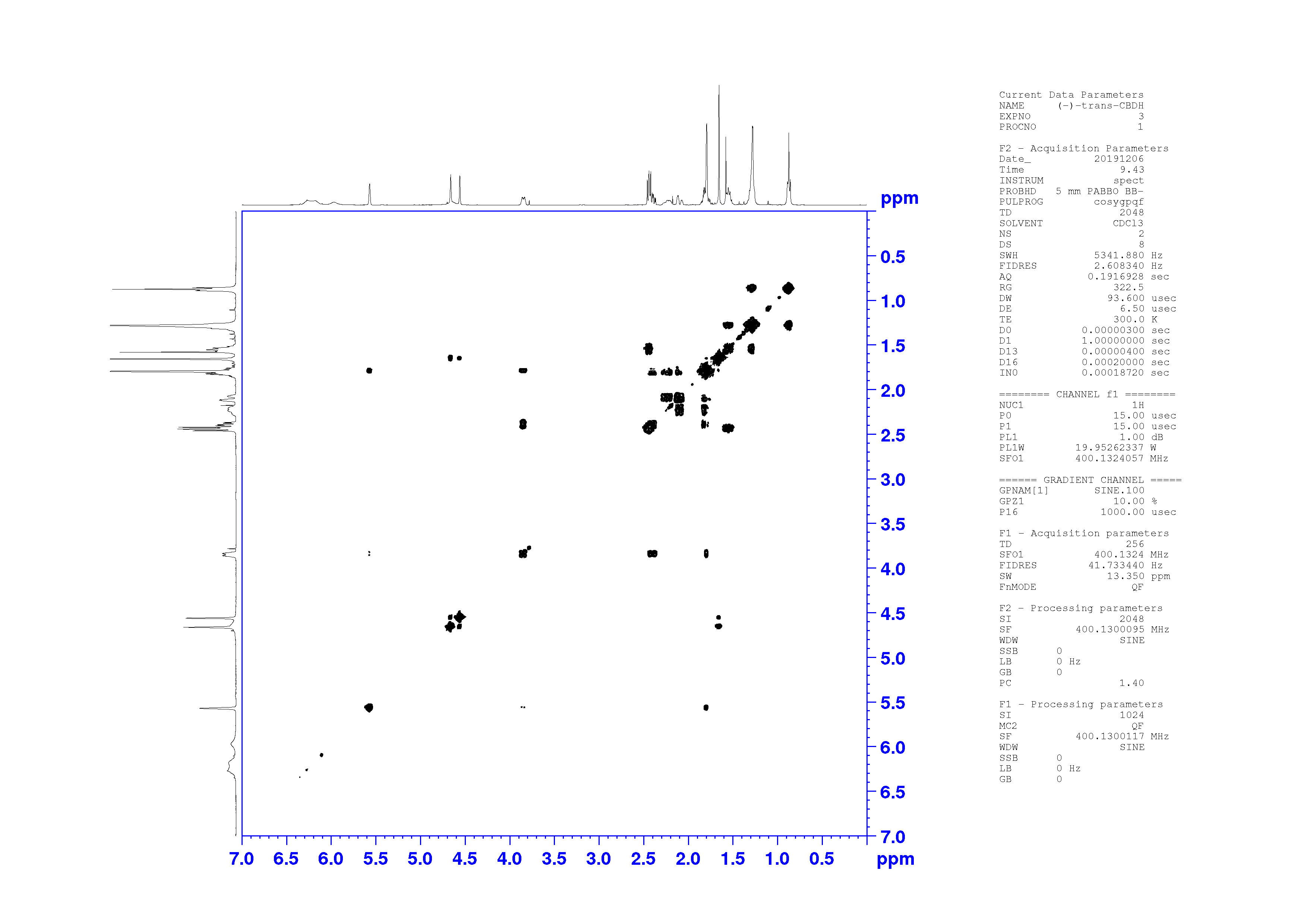 |
| 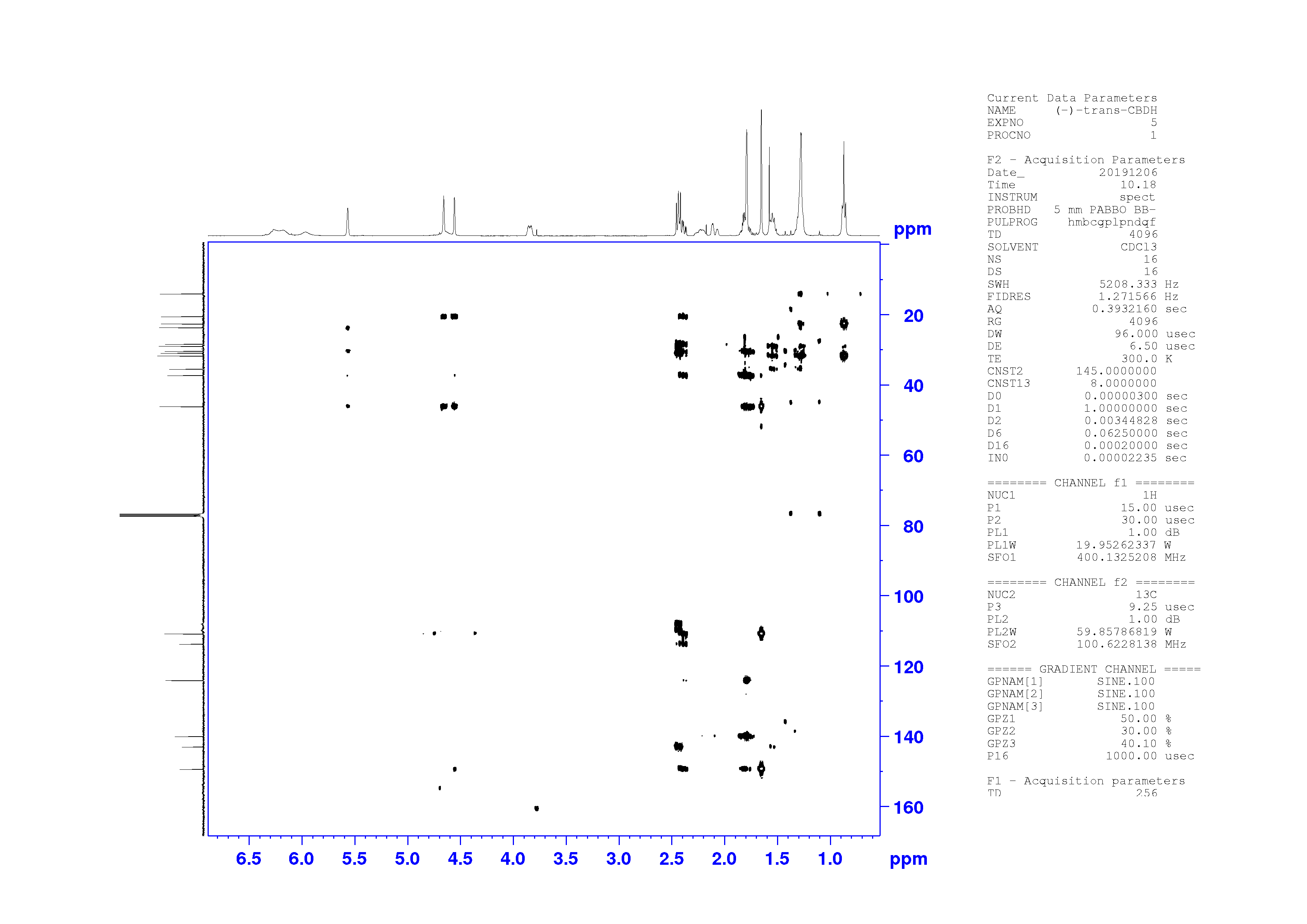 |
| 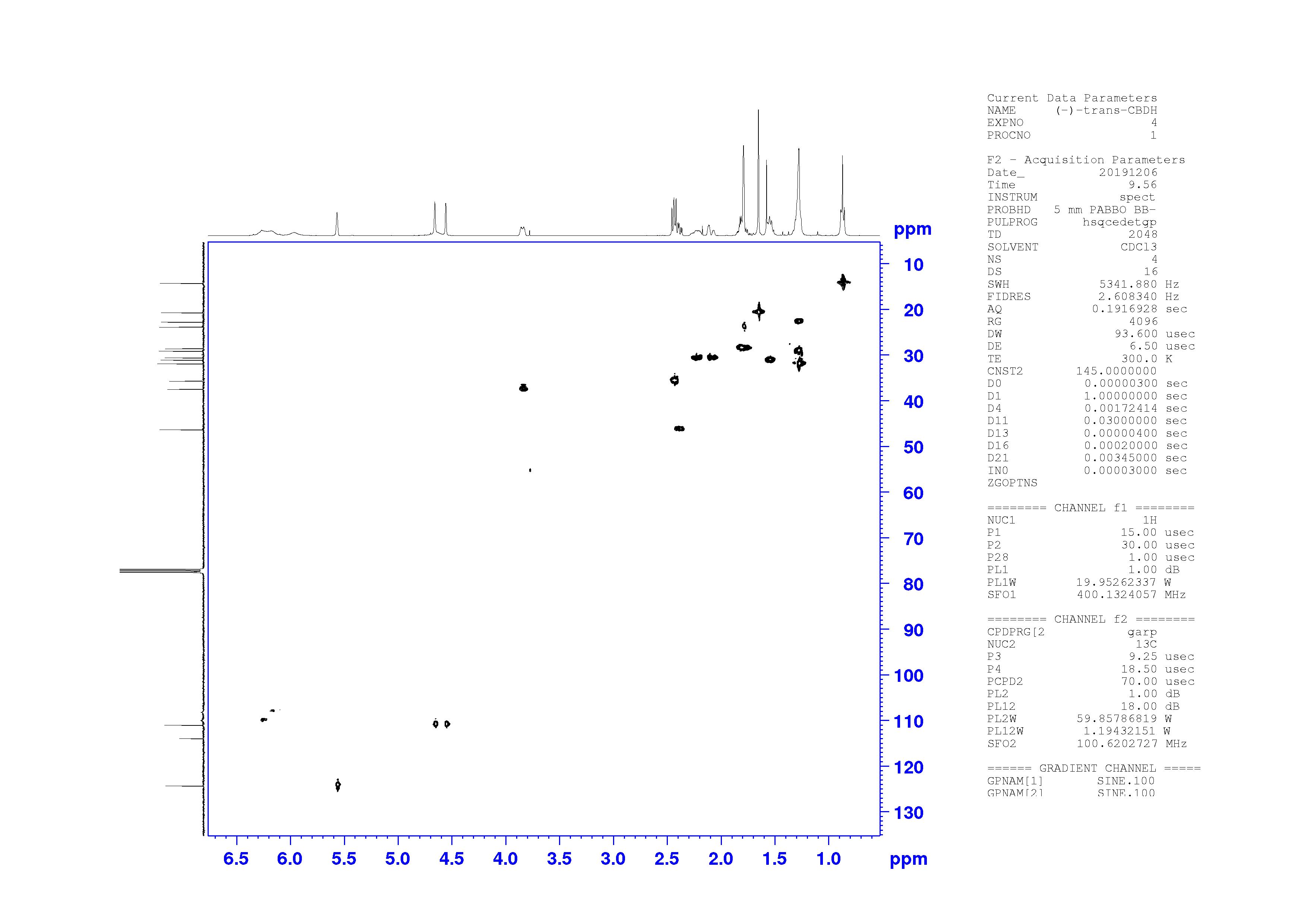 |

| **Figure SI-2.** NMR spectroscopic characterization of synthetic (-)-*trans*-Δ^9^-THCH |
| --- |
| 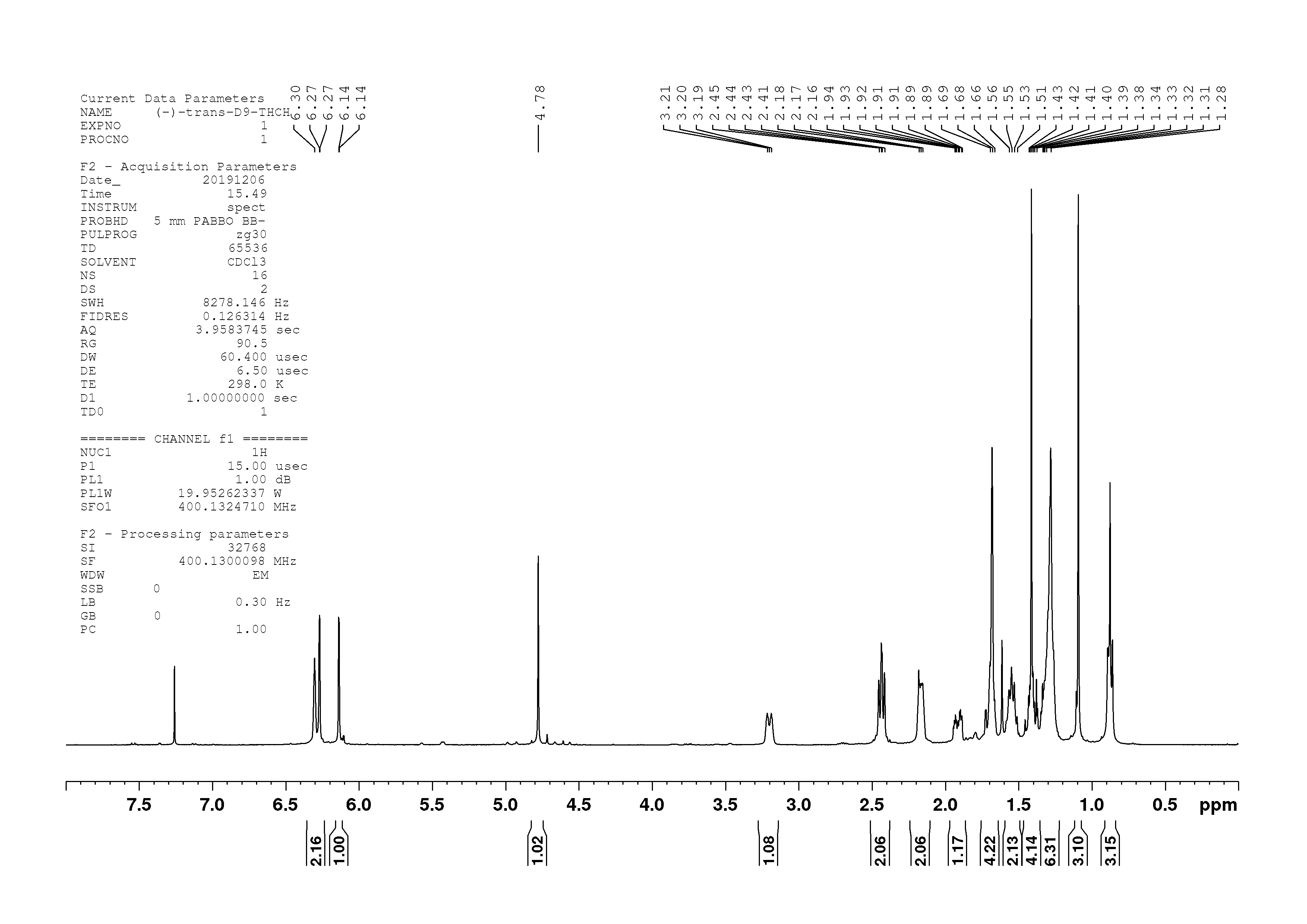 |
| 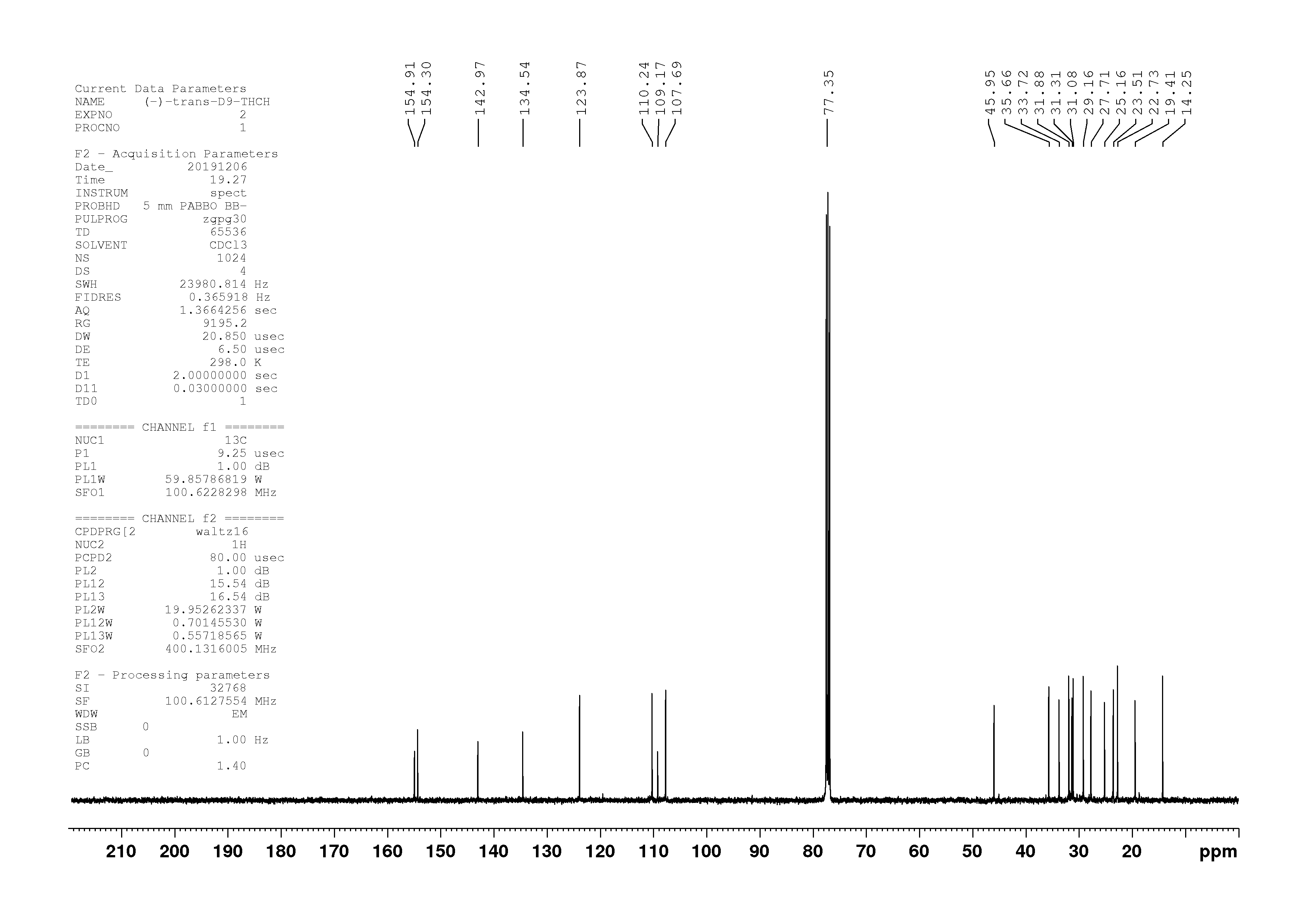 |
| 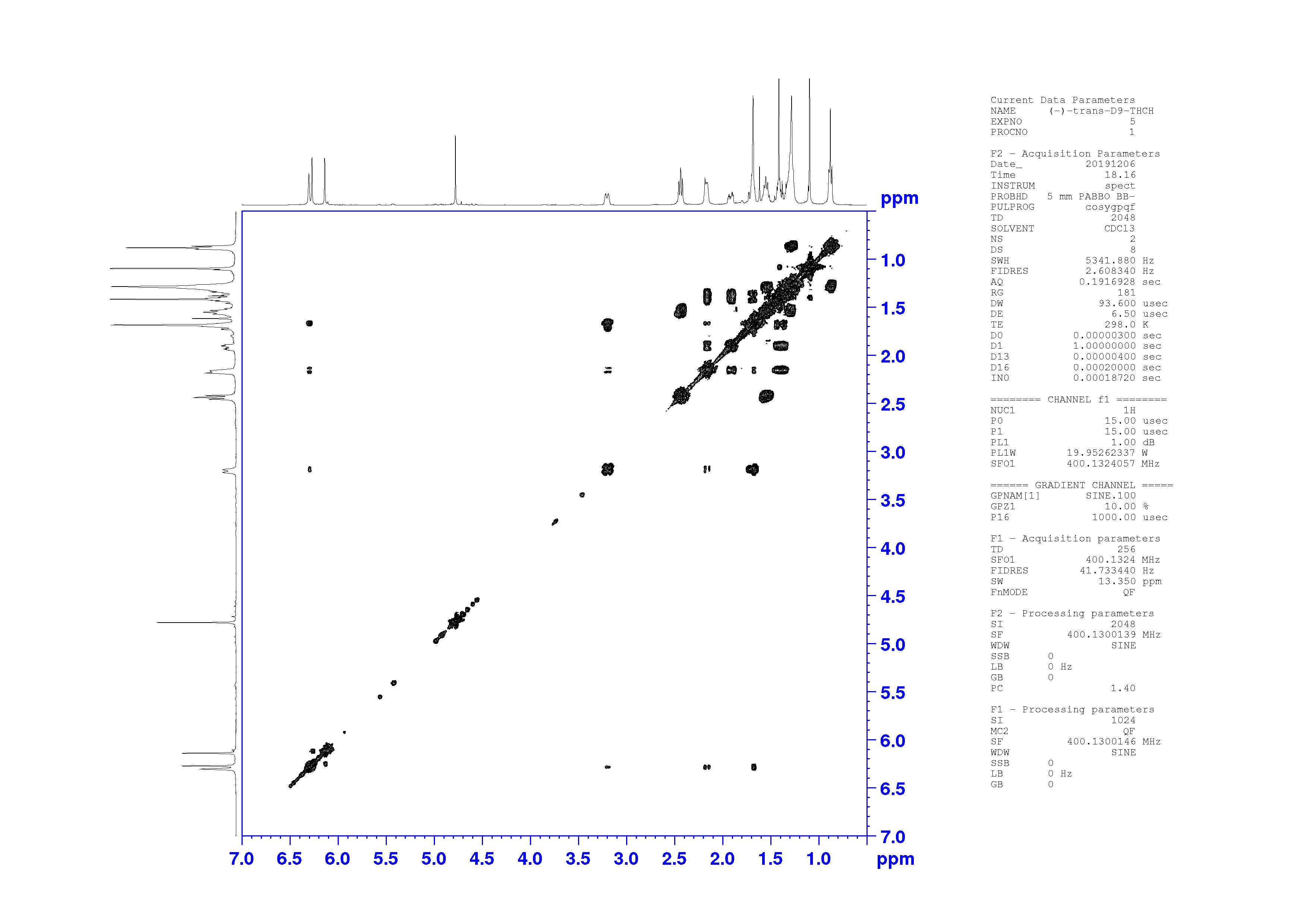 |
| 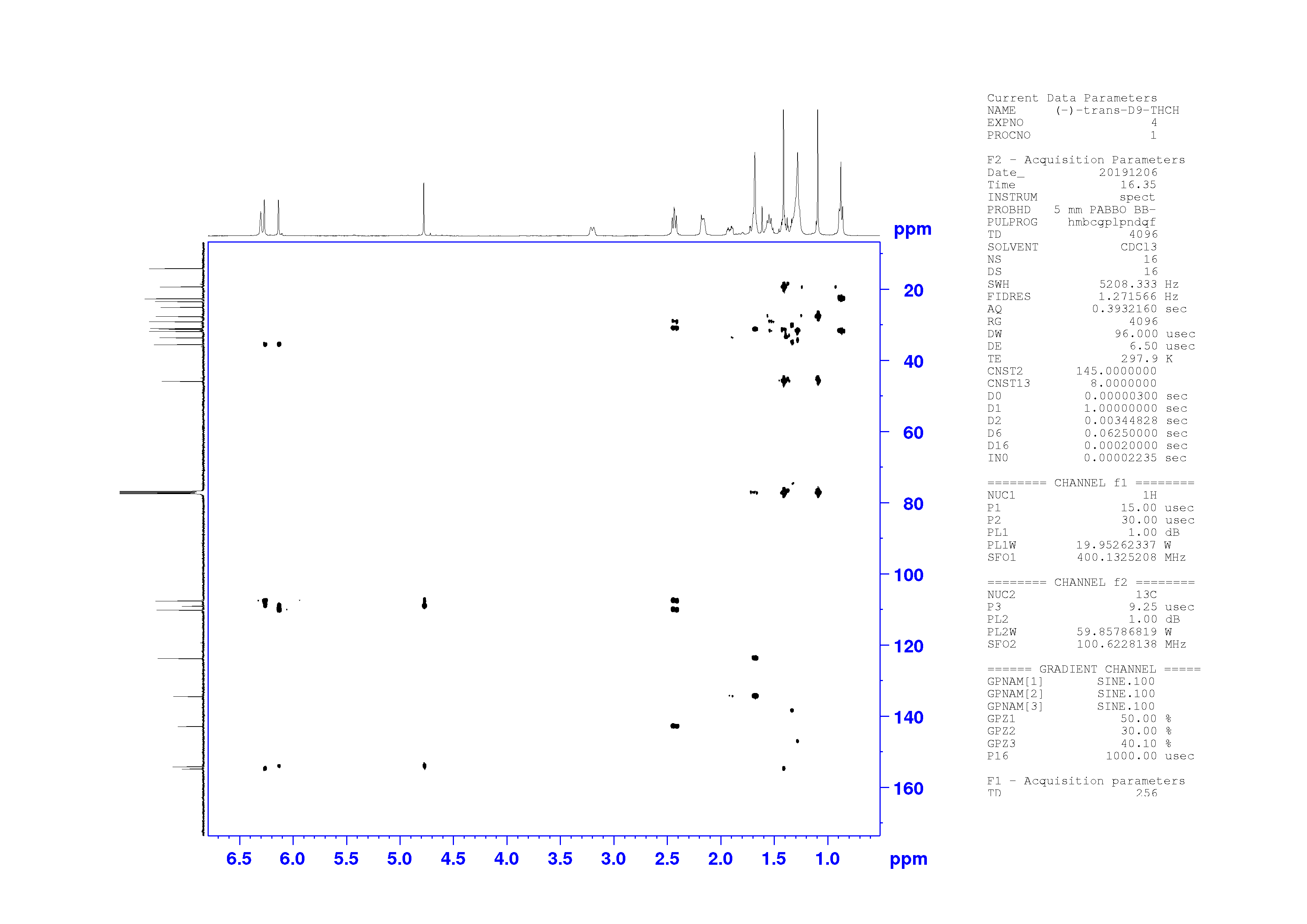 |
| 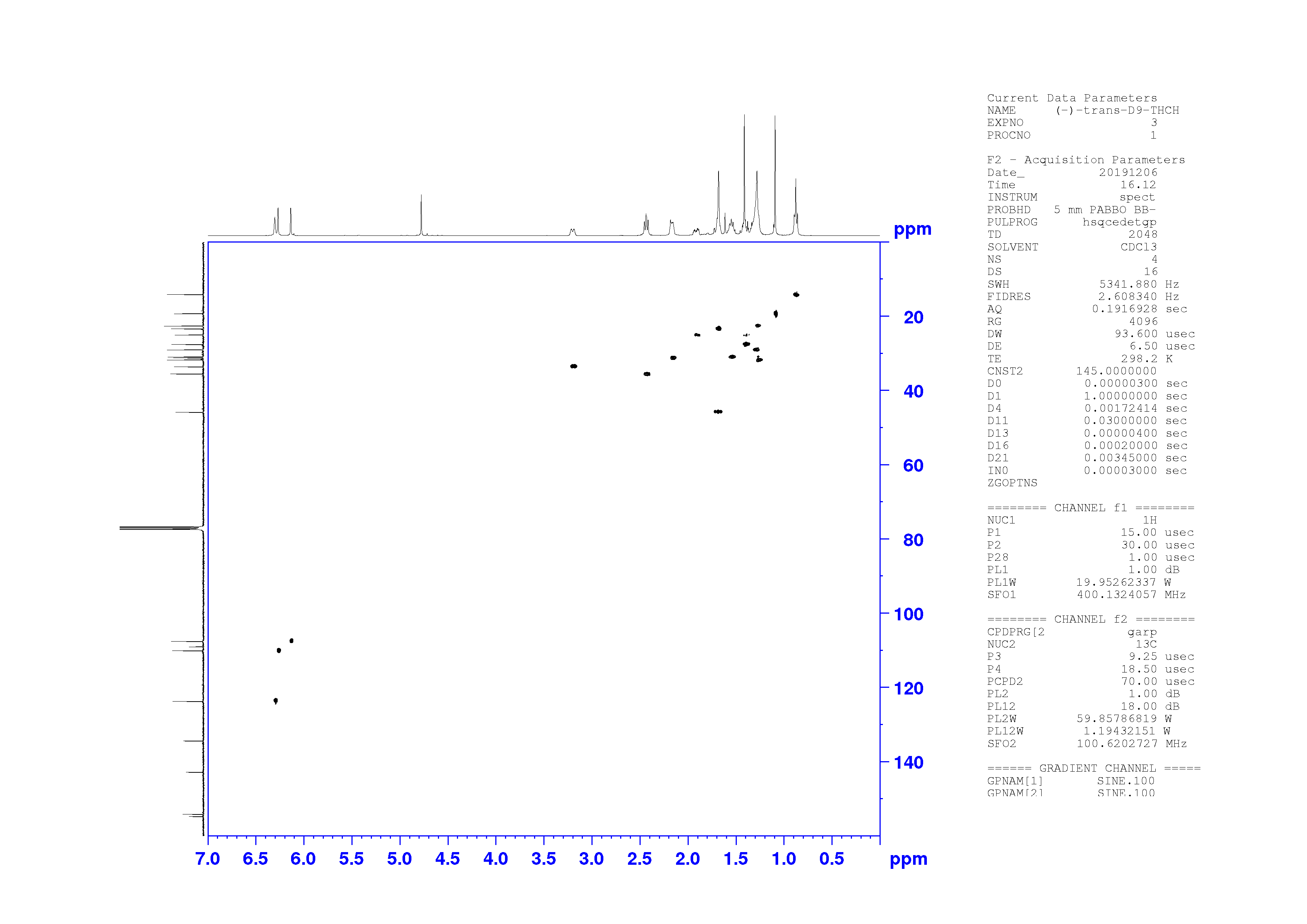 |

| **Figure SI-3.** NMR spectroscopic characterization of synthetic (-)-*trans*-CBDM |
| --- |
| 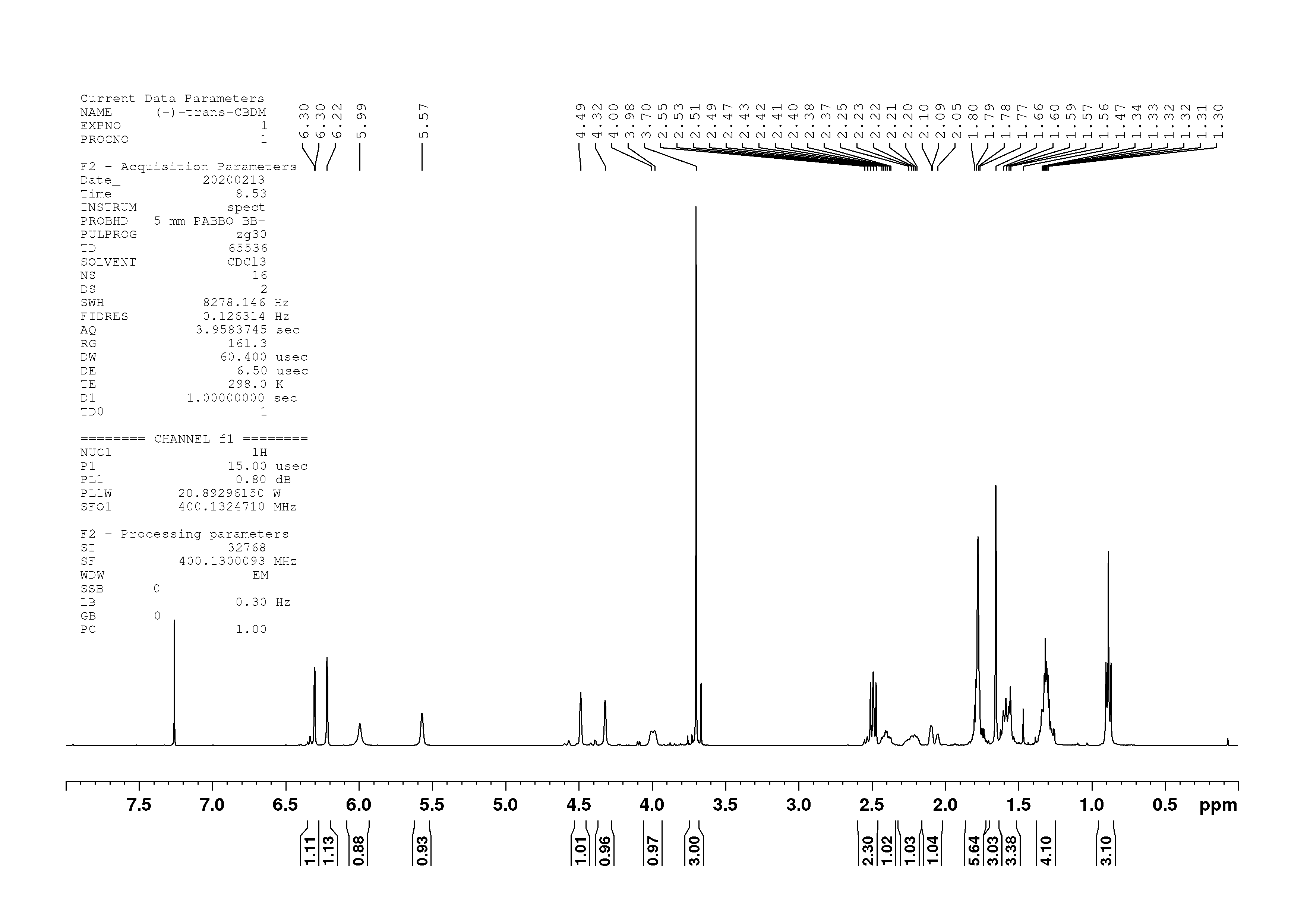 |
| 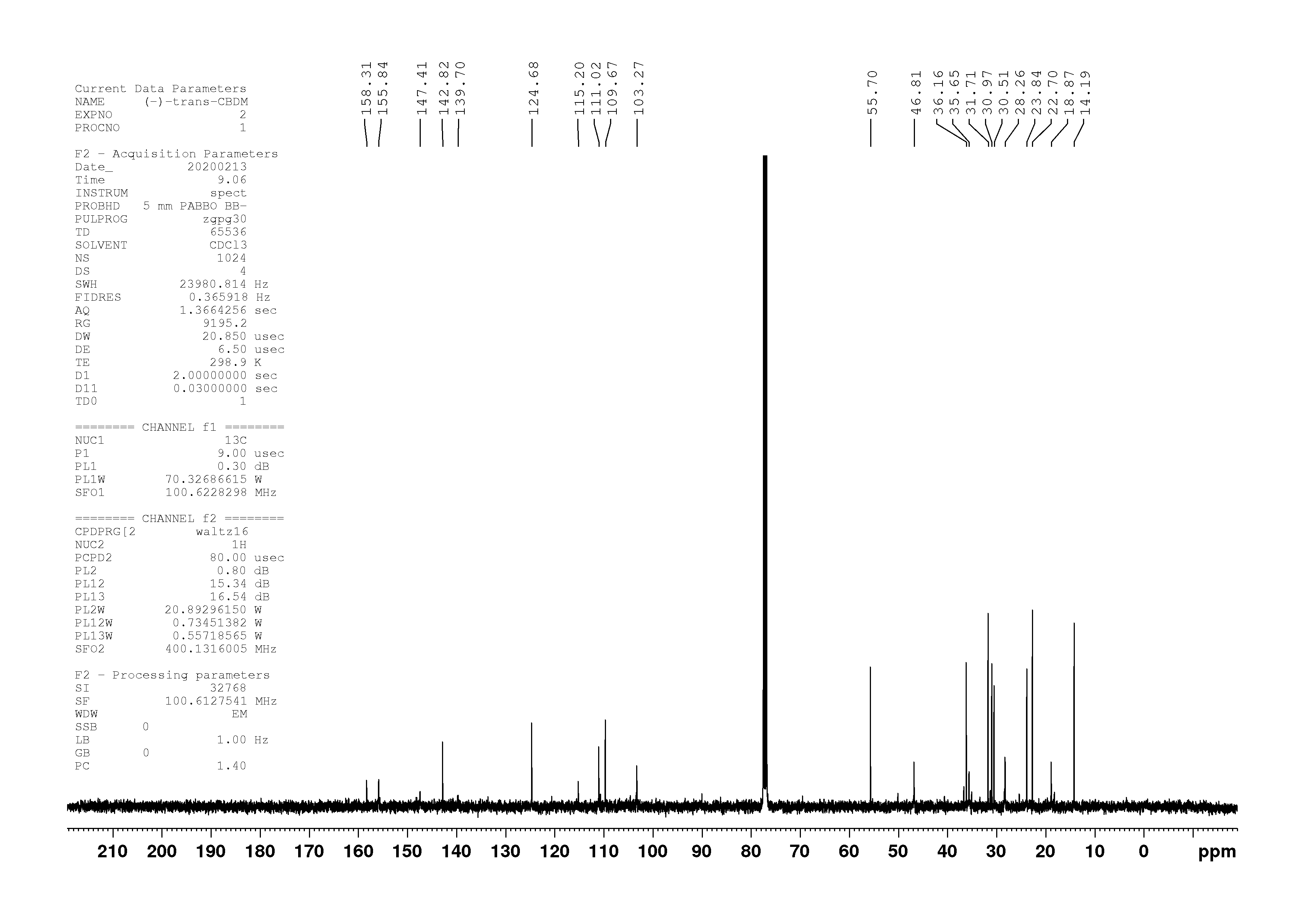 |

| **Figure SI-4.** NMR spectroscopic characterization of synthetic (-)-*trans*-Δ^9^-THCM |
| --- |
| 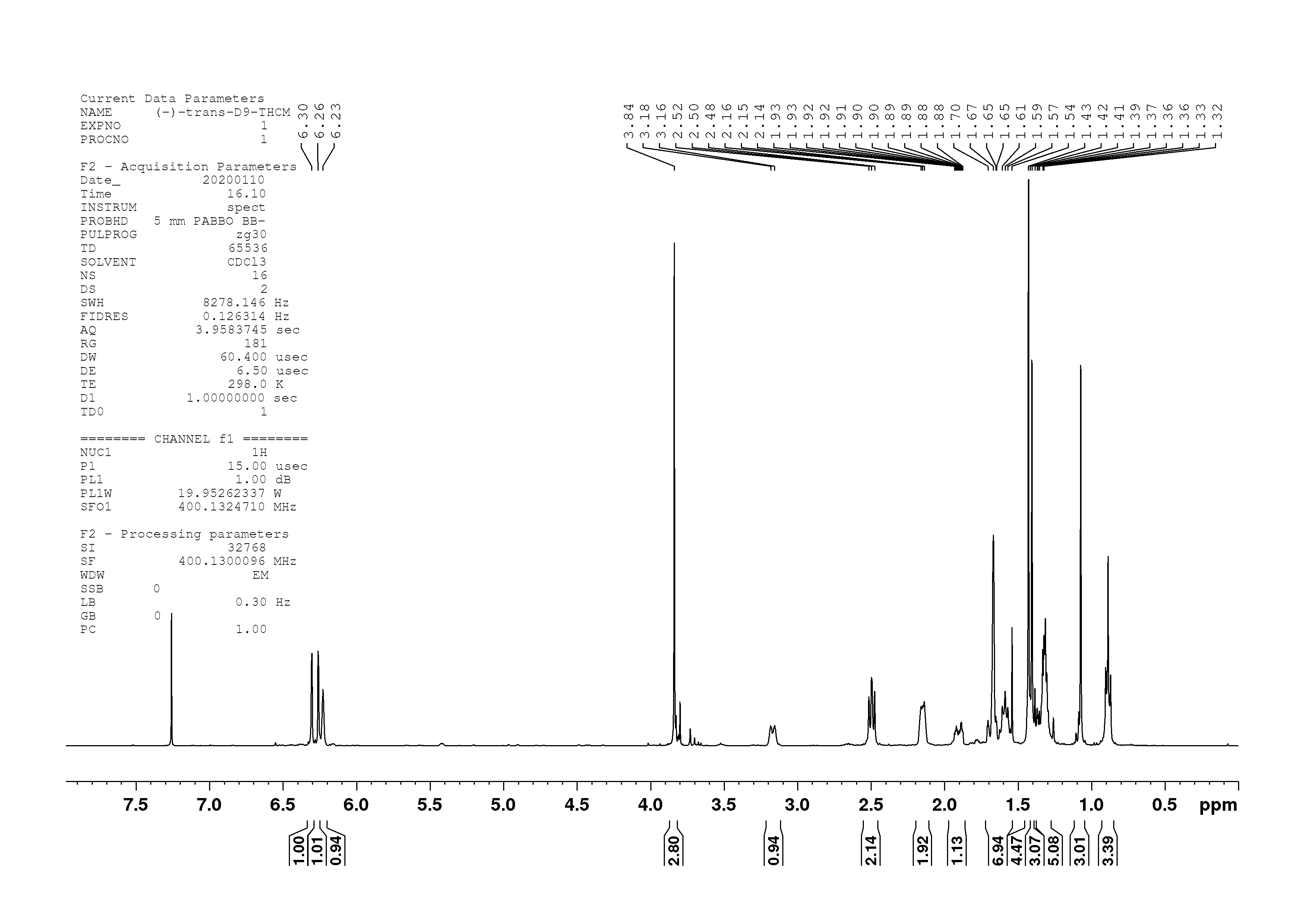 |
| 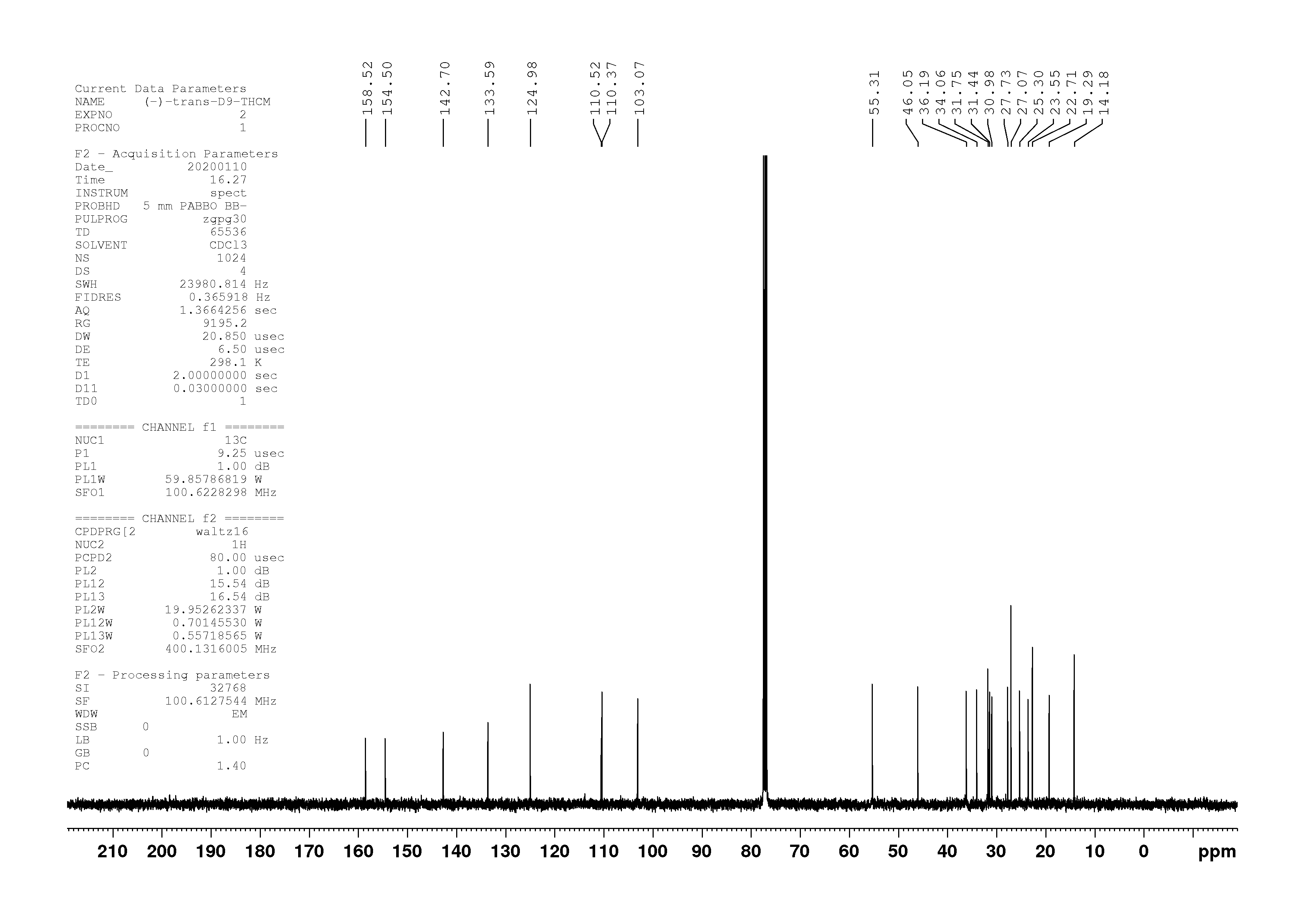 |

| **Figure SI-5.** NMR spectroscopic characterization of synthetic CBGM |
| --- |
| 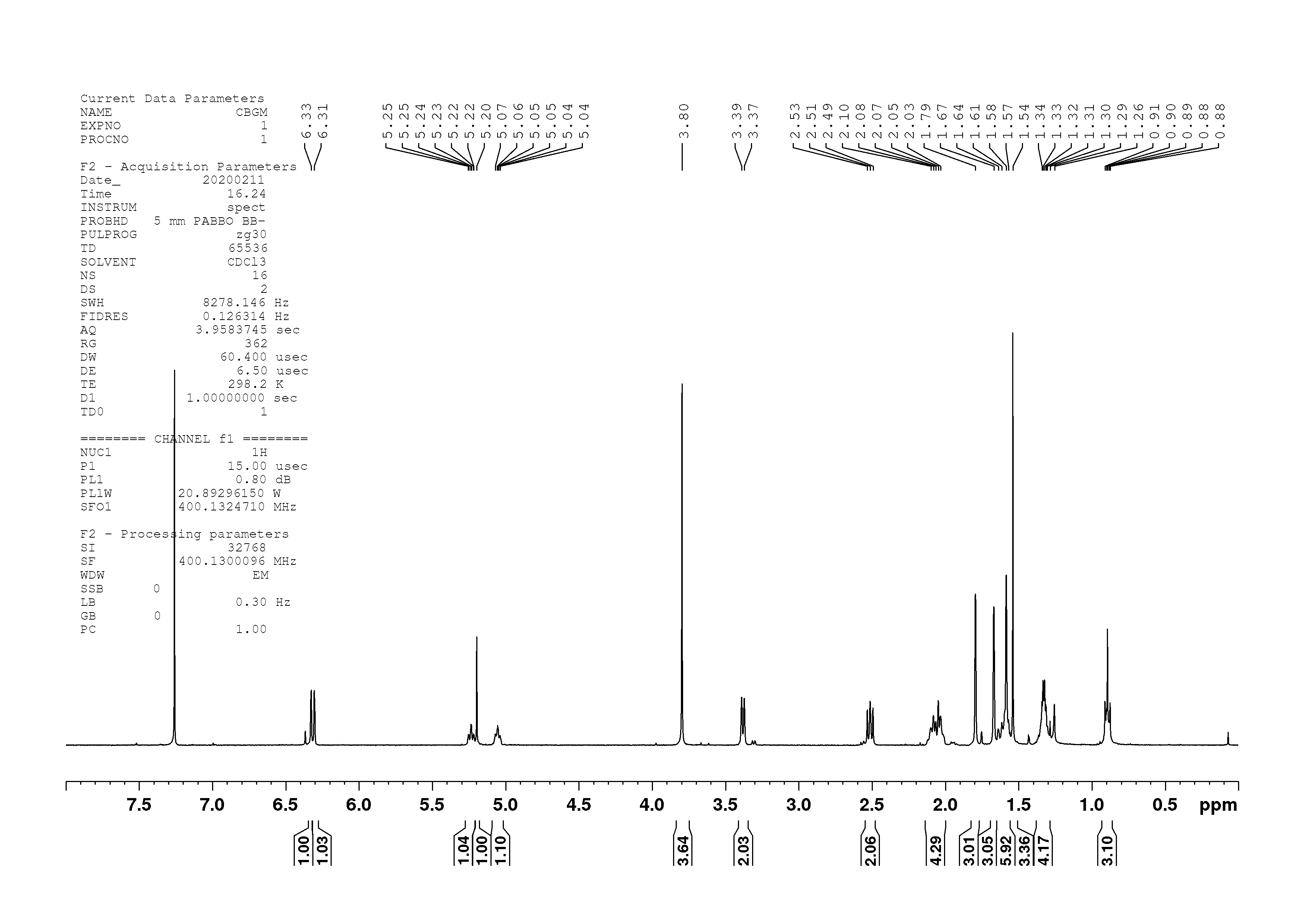 |
| 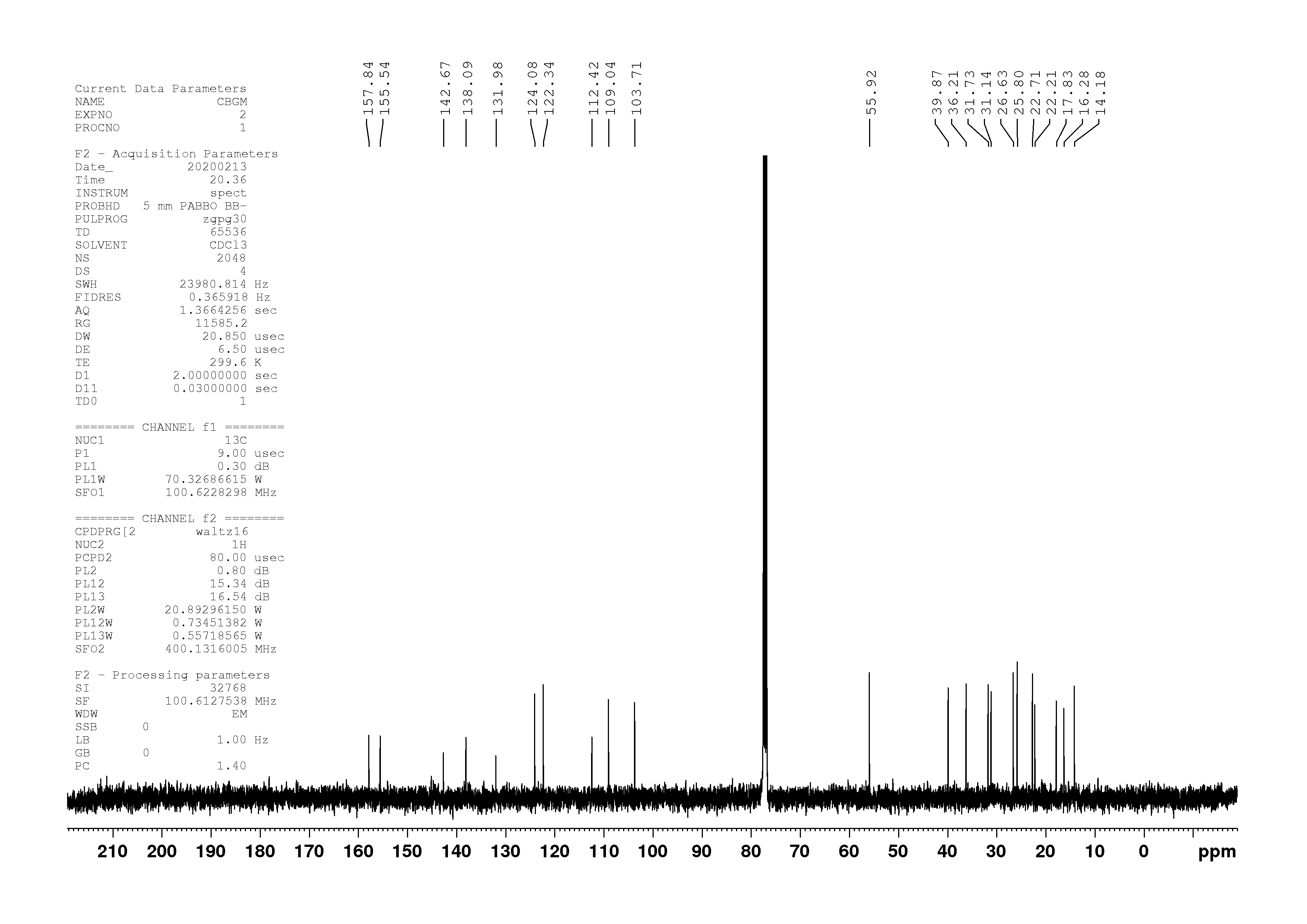 |
| \| **Figure SI-6.** Superimpostion of 1H-NMR and 13C-NMR spectra of synthetic and extracted (-)-trans-CBDH \| \| --- \| \| 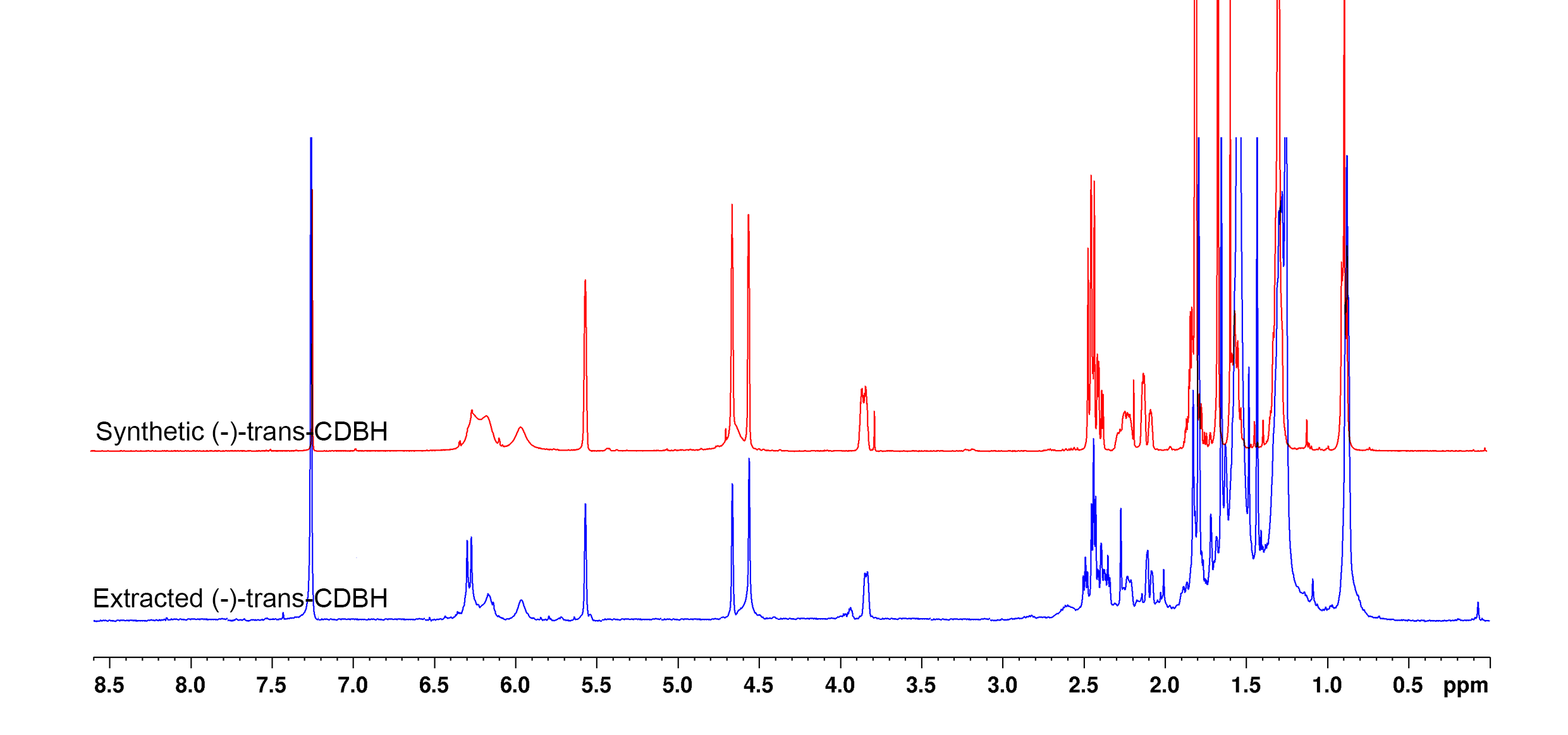 \| \| 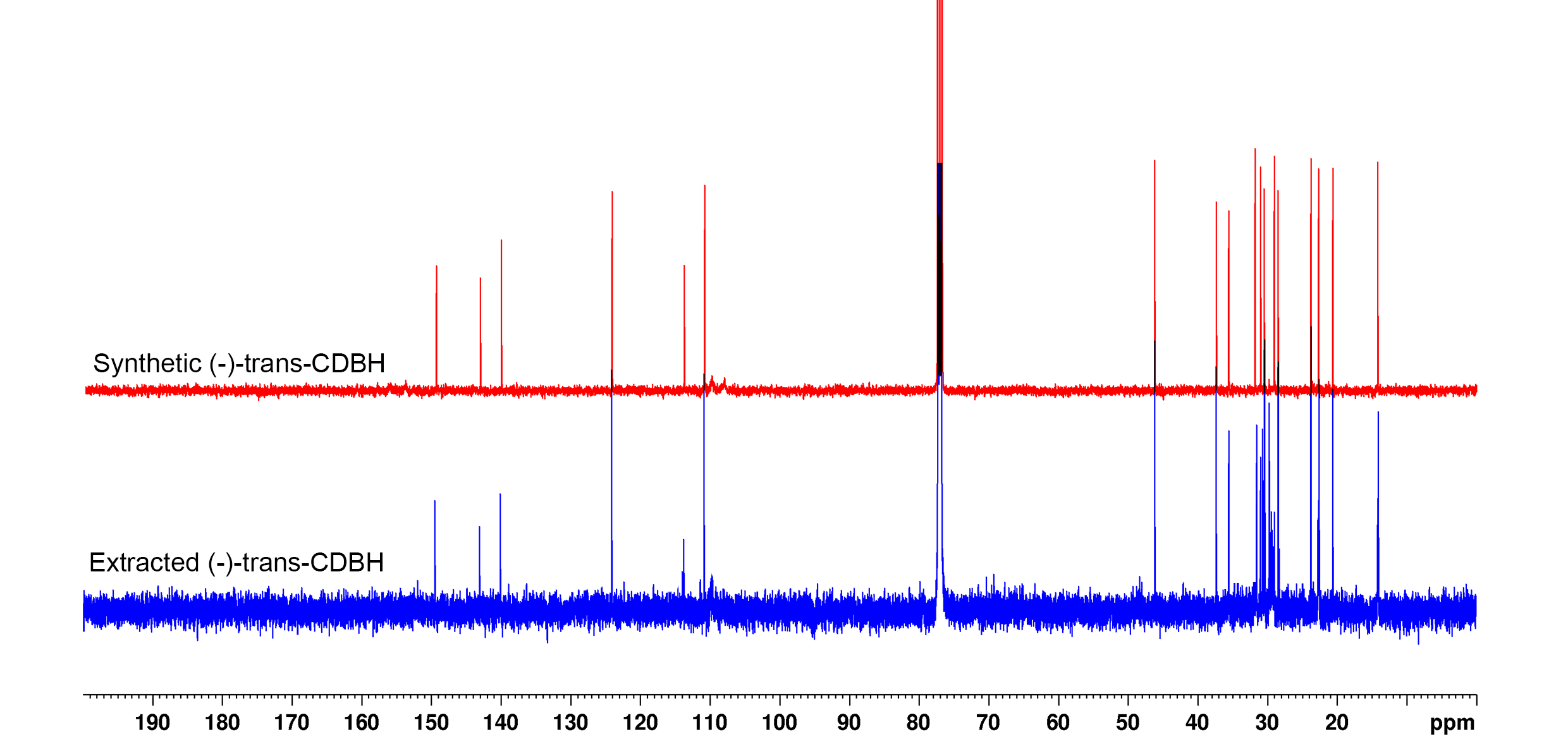 \| |
